# Biomarker guided antibiotic stewardship in community acquired pneumonia: A randomized controlled trial

**DOI:** 10.1371/journal.pone.0307193

**Published:** 2024-08-20

**Authors:** Ruud Duijkers, Hendrik J. Prins, Martijn Kross, Dominic Snijders, Jan W. K. van den Berg, Gwendolyn M. Werkman, Nynke van der Veen, Marianne Schoorl, Marc J. M. Bonten, Cornelis H. van Werkhoven, Wim G. Boersma

**Affiliations:** 1 Department of Pulmonary Medicine, Northwest Hospital, Alkmaar, the Netherlands; 2 Department of Pulmonary Medicine, Zaans Medical Centre, Zaandam, the Netherlands; 3 Department of Pulmonary Medicine, Spaarne Gasthuis, Hoofddorp, the Netherlands; 4 Department of Pulmonary Medicine, ISALA, Zwolle, the Netherlands; 5 Department of Clinical Chemistry, Haematology & Immunology, Northwest Hospital, Alkmaar, the Netherlands; 6 Julius Centre for Health Sciences and Primary Care Health, University Medical Centre Utrecht, Utrecht, the Netherlands; Sant Anna Hospital: Clinica Sant’Anna, SWITZERLAND

## Abstract

**Background:**

In community-acquired pneumonia (CAP), the role of biomarkers to shorten duration of antibiotic treatment has not been firmly established. We assessed the effectiveness of active feedback of treatment algorithms based on procalcitonin (PCT) and C-reactive protein (CRP), compared to standard care, on the duration of antibiotic treatment in patients hospitalized with community-acquired pneumonia (CAP) in non-ICU wards.

**Methods and findings:**

We performed a randomised, open label, parallel group, multi-centre trial in 3 Dutch teaching hospitals. Treatment was guided by a PCT algorithm, CRP algorithm or standard care. Participants were recruited by a member of the study team and randomised at day 2–3 of admission in a 1:1:1 ratio. Treatment was discontinued upon predefined thresholds of biomarkers that were assessed on admission, day 4 and days 5–7 if indicated. The primary outcome was total days on antibiotic treatment until day 30. In total 468 participants were included in this study. The median days on antibiotics (IQR) was 7 (IQR 7–10) in the control group, 4 (IQR 3–7) in the CRP group (rate ratio (RR) of 0.70, 95% CI 0.61–0.82 compared to standard care; p <0.001), and 5.5 (IQR 3–9) in the PCT group (RR of 0.78, 95% CI 0.68–0.89 compared to standard care; p <0.001). New antibiotics within the first 30 days were prescribed to 24, 23 and 35 patients in standard care, CRP and PCT groups, respectively. The hazard ratio for a new prescription in patients in the PCT group compared to standard care 1.63 (CI 0.97–2.75; p = 0.06). No difference in time to clinical stability or length of stay was found.

**Conclusions:**

A strategy of feedback of CRP-guided and PCT-guided treatment algorithms reduced the number of days on antibiotic in the first 30 days after hospital admission in non-ICU wards for CAP. The study was not powered to determine safety of shortening duration of antibiotic treatment. (NCT01964495).

## Introduction

Community-acquired pneumonia (CAP) is an important cause of death worldwide [1]. In Europe 3.3 million people develop CAP per year, of whom 20–50% need hospital admission. The annual costs associated with CAP in Europe amount to ~€10.1 billion, with inpatient care accounting for €5.7 billion and treatment accounting for ~€0.2 billion [2, 3].

Guidelines recommend antibiotic courses of five to 21 days, depending on severity of illness, causative pathogen, clinical response and type of antibiotic used [4–6]. Yet, in daily practice physicians tend to treat longer than recommended, especially in patients with significant comorbidities, in patients who fail to respond rapidly on antibiotic treatment and in patients with severe CAP [7–10].

This underlines the need for guidance to shorten the duration of antibiotic treatment without compromising patient safety.

Biomarkers have been proposed as objective means to tailor antibiotic treatment in patients with CAP. PCT is the most studied biomarker, which seems useful to withhold or discontinue antibiotics in patients with acute respiratory infections, including CAP, without an increase in treatment failure or mortality [11, 12]. However, concerns have been raised regarding patient selection in clinical trials, non-adherence to PCT algorithms by treating physicians and usefulness of PCT in patients with atypical pathogens or renal failure [13, 14].

In the Netherlands C-reactive protein (CRP) is the most commonly used biomarker in patients hospitalized with CAP. Results from two observational studies in patients with CAP suggested that CRP might aid the clinical decision-making process [15, 16].

We, therefore, performed a randomised controlled multi-centre trial to quantify the effects of a CRP and a PCT based algorithm, compared to routine care, on the duration of antibiotic treatment duration in hospitalized patients with CAP.

## Materials and methods

### Trial design and oversight

This is a multi-centre randomised controlled parallel group open-label trial involving patients hospitalized with CAP in non-ICU hospital wards of three teaching hospitals in the Netherlands (The Northwest hospital Alkmaar, ISALA clinics Zwolle and the Slotervaart Hospital in Amsterdam).

Prior to the trial, all participating centres were familiar with CRP measurements in routine care. None of the participating centres used PCT in routine care.

The study protocol was approved by the Medical Ethics Committee associated with the Northwest hospital (METC- registration: M013-031, CCMO-registration NL44806.094.13) and is in full compliance with the Helsinki declaration. The study protocol was registered in the clinicaltrials.gov database. (NCT01964495).

Eligible patients were approached for written informed consent twice. At the time of admission a short written informed consent was obtained to collect a blood sample for determination of PCT levels, as this was not part of standard care. At day two or three prior to randomisation a full written consent was obtained.

Recruitment started December 5, 2013 in the Northwest hospital, followed by the Slotervaart Hospital in July 2014 and finally the ISALA clinics in March 2015. Recruitment was completed in all hospitals in October 28, 2016. The authors vouch for the quality of the data collection and analysis.

### Participants

**Fig 1:**
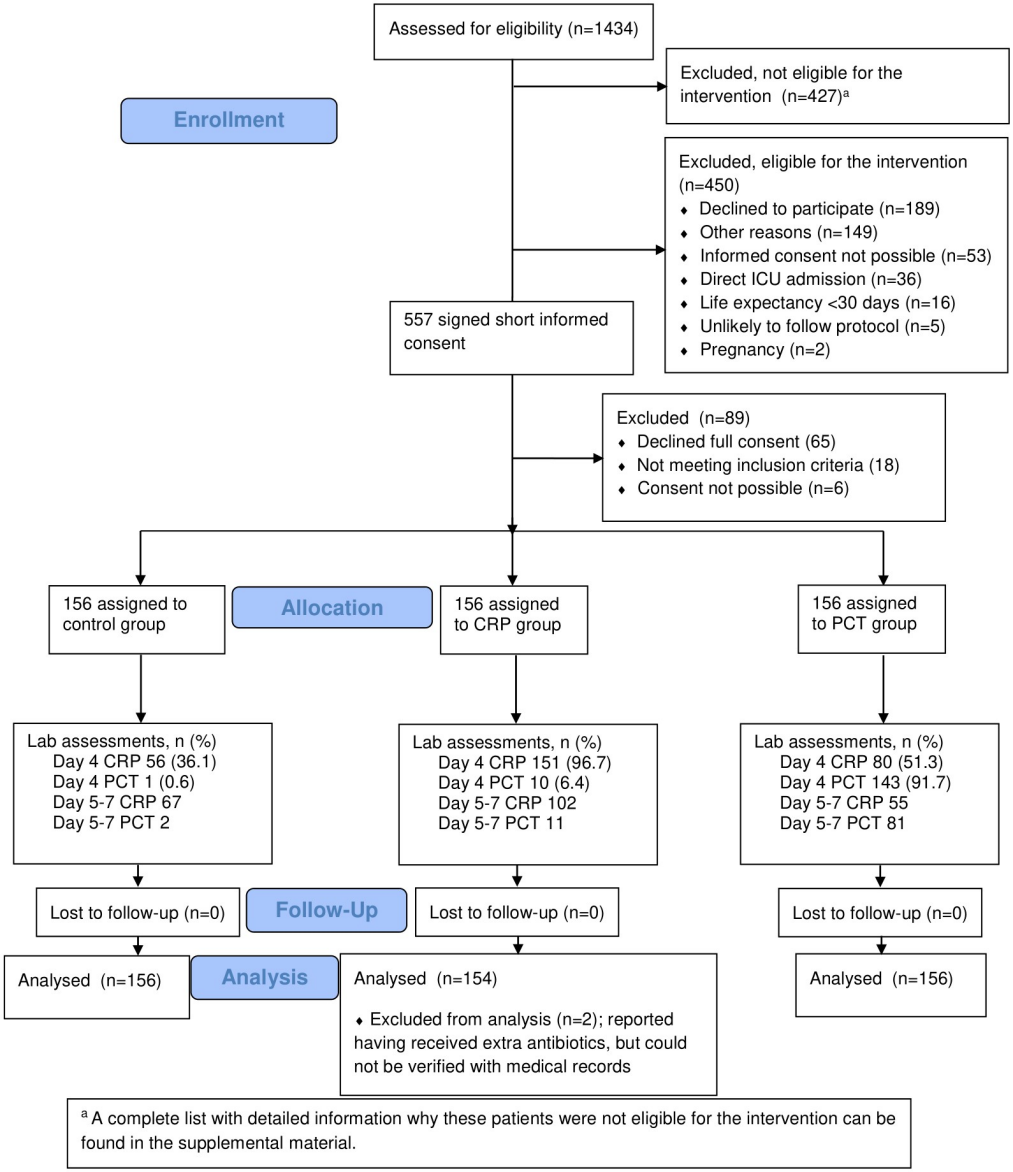
Consort flow diagram.

All adult patients with a clinical diagnosis of CAP made by the attending physician were assessed for eligibility. The attending physician made the decision whether or not the patient required hospitalization or ICU admission based on routine care. Patients with radiologically confirmed CAP admitted to a non-ICU ward without severe immunosuppression, active neoplastic disease, obstruction pneumonia, or aspiration pneumonia, were eligible for the study (see S1 Appendix for full criteria). If another diagnosis was established prior to randomisation and antibiotic treatment was stopped, patients were not randomised and excluded from analysis (Fig 1).

### Simple size calculation

Sample size calculation was done using the program G-power. We assumed a mean (SD) treatment duration of 8.8 (SD ±5.9) days based on routine clinical practice in the Northwest clinics and of 6.8 (SD ±4.8) days when using the CRP and PCT-based algorithms. Using a two-sided α of 0.025 (corrected for multiple testing using the Bonferroni Holm method) and β of 0.20 resulted in a total of 139 patients required per group assuming a normal distribution of the primary endpoint and of 146 patients per group with non-normal distribution. We included a total number of 156 patients per group to account for loss to follow-up, deaths etc. which amounts to a total number of 468 patients.

### Randomisation

We compared patients treated according to current guidelines (control group) with patients in whom antibiotic treatment was guided by serum PCT levels (PCT group) or by serum CRP levels (CRP group). Randomisation was performed on day two or three of admission in a 1:1:1 ratio by means of block randomisation using blocks of 30 patients at a time and one final block of 18. Blocks were generated by an independent statistician. Each centre was assigned a block and upon completion was assigned the next block of 30. Patients were allocated to one of the three groups by sealed opaque envelopes. After randomisation no masking was performed.

### Interventions

Baseline assessment included clinical data, vitals, comorbid conditions, medication and routine blood tests. Chest x-rays were reviewed by attending physicians, who also decided whether or not to start empiric CAP treatment.

Standard microbiological tests consisted of blood cultures, sputum culture (if possible), urinary antigen tests for pneumococci and legionella and an oropharyngeal swab for multiplex PCR for atypical and viral pathogens (RespiFinder^®^ 2SMART version 2.2 en 2.3). All results were reported to treating physicians according to routine practice.

Patients were treated according to Dutch national guidelines for the first three days of admission (6). The first day of admission was defined as day one, even if a patient was admitted in the evening and only received one dose of antibiotics. Consequently, if antibiotics were stopped on day four, duration of treatment was counted as three days.

In the control group the duration of antibiotic therapy was based on national guidelines and the time of stopping antibiotics was left to the discretion of the attending physician. In all study groups, physicians were free to order routinely available diagnostic tests, during the patients’ hospital stay.

CRP analysis was performed using C-reactive protein reagent and the Beckman Synchron DxC 800 analyzer (Beckman Coulter Inc., Brea, California, USA). Serum samples were analyzed within 2 hours after collection. PCT analysis was performed using the Vidas B.R.A.H.M.S. PCT assay and the Vidas immunoanalyzer (BioMerieux, Marcy l’Etoile, France). Serum samples were analyzed within 2 hours after collection.

CRP and PCT were determined on day 1 for all patients and then in the intervention groups on day four. If the day-4 level was below the threshold value (below 100 mg/L **and** a reduction to below 50% of the initial value for CRP and below 0.25 μg/L **or** a reduction to below 10% of the initial value for PCT) antibiotics were discontinued. If the level was not low enough to discontinue antibiotics, CRP or PCT was determined daily until the threshold was reached until day 7 at the latest. If patients were discharged before antibiotics were stopped, additional blood samples were collected during outpatient visits or home visits. These patients were informed about continuing or stopping antibiotics by a member of the research staff, who are all physicians of the pulmonology department. In case of any doubt whether or not antibiotics could safely be discontinued when the patient was still exhibiting symptoms, this decision was left up to a senior member of the pulmonology staff.

All outcomes were assessed at an outpatient visit at day 30±2.

CRP threshold values were derived from the CAPISCE study, a clinical trial in patients with CAP with daily measurement of biomarkers during the first week of admission [17]. PCT threshold values were derived from the study performed by Christ-Cain et al. [18].

Attending physicians regularly received in-person training on study protocol and were allowed to deviate from protocol for safety reasons. Reasons for protocol deviations were documented. All biomarker results were actively checked by a member of the research staff and communicated to the treating physician.

### Outcomes

The primary endpoint was total number of days on antibiotic treatment until day 30. This includes IV and oral treatment. Secondary endpoints consisted of new antibiotic prescriptions, length of stay, time to clinical stability and all-cause mortality, all with a time-window of 30±2 days from admission. All outcomes were assessed and recorded by a designated member of the research team in each centre.

New antibiotic prescriptions were defined as: any broadening, prolongation beyond originally planned treatment duration (including prolongation of treatment beyond the recommended duration as determined by the biomarker algorithms in the intervention arms), or restarting of antibiotic treatment during the intervention period (day four and onward). Reasons for new prescriptions were documented. Only the first new prescription was counted as an event. Originally in our protocol the term treatment failure was used instead of new antibiotic prescriptions, but the latter is more accurate so is used throughout this manuscript instead. The definition of this term remains unchanged.

Clinical stability was defined according to the criteria mentioned in the IDSA/ATS CAP guideline [5]. This list can be found in the S2 Appendix.

### Statistical analysis

All data was analysed using IBM SPSS statistics version 20 for Windows and R statistics.

Initially we planned to analyse the primary endpoint by comparing means/medians with standard parametric or non-parametric tests, however upon completion of our trial we realized that a negative binomial model with robust standard errors would be more appropriate as it yields an effect size rather than a p-value, so we changed our analysis accordingly. We used robust standard errors because due to our study design most patients are either treated for three days or seven days, which violates the distributional assumption of a negative-binomial model. The primary outcome is reported as Rate Ratios (RR) with 95% confidence intervals, reflecting the relative change in the number of calendar days on antibiotic treatment.

Length-of-stay was analysed using a Cox proportional hazards model and a competing events regression model with death as a competing variable using R statistics.

All other outcomes were assessed using a Cox proportional hazards model and reported with hazard ratios and 95% confidence intervals. Any patient that died during hospital admission was censored in the analysis for length of stay. Patients that did not meet criteria for clinical stability prior to discharge or died during hospital admission were censored in the analysis for time to clinical stability.

Patients were analysed according to the allocated intervention, i.e. using an intention-to-treat approach.

## Results

### Participants

1434 patients were screened for eligibility and 895 met the in- and exclusion criteria, of which 557 signed the short informed consent for assessing PCT values at the time of admission. Of these, 468 were randomised, as 65 declined full informed consent, 18 did not meet in- and exclusion criteria on admission, four patients could not consent due to delirium and inability to reach a legal representative and in two patients palliative care was started prior to informed consent. Reasons for non-inclusion and non-randomisation are detailed in Fig 1. Baseline characteristics are outlined in Table 1. Results of all microbiological tests appear in the S1 Table. In 297 (63.5%) patients a potential pathogen could be identified. An overview of co-infections appears in the S2 Table.

**Table 1:** Baseline characteristics on admission.

	Control group (n = 156)	CRP group (n = 156)	PCT group (n = 156)
Age in years, means ± SD	67 ± 16	68 ± 15	67 ± 15
Male sex, no. (%)	94 (60.3)	92 (59.0)	87 (55.8)
Smoking status			
Former smoker, no. (%)	76 (52.8)	84 (58.3)	81 (54.4)
Current smoker, no. (%)	42 (29.2)	33 (22.9)	37 (24.8)
Pack years of current and former smokers, median (IQR)	30 (16–42.3)	32 (13–48)	30 (16.5–50)
Antibiotic pre-treatment, no. (%)	39 (25.3)	38 (24.7)	31 (20.4)
Coexisting illnesses, no. (%)			
Congestive heart failure	20 (12.8)	21 (13.5)	19 (12.3)
Cerebrovascular disease	8 (5.1%)	16 (10.3%)	9 (5.8%)
Chronic renal disease	9 (5.8%)	5 (3.2%)	9 (5.8%)
Liver disease	1 (0.6%)	1 (0.6%)	2 (1.3%)
Diabetes Mellitus	16 (10.3)	21 (13.5)	22 (14.1)
COPD	46 (32.2)	51 (35.4)	53 (35.6)
Charlson comorbidity index, median (IQR)	1.00 (0–2)	1.00 (0–2)	1.00 (0–2)
Examination			
Body temperature, °C, means ± SD	38.3 ± 1.0	38.2 ± 1.1	38.3 ± 1.0
Oxygen saturation, %, means ± SD	93.9 ± 3.6	93.6 ± 4.4	93.2 ± 4.0
Supplemental Oxygen, no (%)	104 (66.7)	106 (67.9)	105 (67.3)
Heart rate, beats/min, means ± SD	97.5 ± 19.8	99.7 ± 20.5	101.0 ± 20.2
Systolic blood pressure, mm Hg, means ± SD	131.6 ± 21.6	132.2 ± 22.6	132.1 ± 22.2
Diastolic blood pressure, mm Hg, means ± SD	74.0 ± 14.4	74.6 ± 13.7	75.0 ± 15.6
Laboratory findings on day 1			
Procalcitonin (μg/L), median (IQR) ^a^	0.475 (0.120–4.053)	0.510 (0.120–5.100)	0.555 (0.113–4.825)
C-reactive protein (mg/L), median (IQR)	182 (96–249)	162 (80.5–265)	154 (85–257)
Leukocyte count (x 10^9^/L), median (IQR)	14.05 (11.23–17.45)	12.6 (9.70–17.58)	13.8 (10.20–18.10)
PCT < 0.25 μg/L, no. (%)	59 (37.8)	59 (37.8)	64 (41.0)
CRP ≥ 100	114 (73.1)	110 (70.5)	109 (69.9)
CRP 50–100 mg/L, no. (%)	17 (14.9)	26 (16.7)	28 (17.9)
CRP < 50 mg/L, no. (%)	25 (16.0)	20 (12.8)	19 (12.2)
Imaging, no. (%)			
Pleural effusion	33 (21.2)	24 (15.5)	29 (18.6)
Multilobar pneumonia	44 (28.2)	47 (30.1)	44 (28.2)
CURB-65 score, median (IQR)	1 (0–2)	1 (1–2)	1 (1–2)
CURB-65 score ≥3 no. (%)	21 (13.5)	24 (15.4)	22 (14.1)
Empiric regime covering atypical pathogens, no (%)	48 (31)	61 (39)	37 (24)

^a^ The conversion factor for procalcitonin is: μg/L

* 0.161 = nmol/L.

### Interventions

On admission CRP and PCT were determined in 468 and 464 patients, respectively. At day four, CRP was determined in 56 (36.1%) in the control group, in 151 (96.7%) in the CRP group and in 40 (25.6%) in the PCT group. At day four, PCT was determined in 1 (0.6%) in the control group, in 10 (6.4%) in the CRP group and in 143 (91.7%) in the PCT group. Follow-up CRP measurements after day four were performed in 67, 102 and 55 patients in the control, CRP and PCT group, respectively. Follow-up PCT measures after day four were performed in 2, 11 and 81 patients in the control, CRP and PCT group, respectively. All patients complied with sample collection. In case of logistical errors, e.g. when samples for biomarker testing were not ordered, or samples were lost during transportation or processing, an attempt was made to collect another sample in time. If that failed, antibiotics were continued and a sample was taken the next day, up until day 7.

### Primary outcome

**Fig 2:**
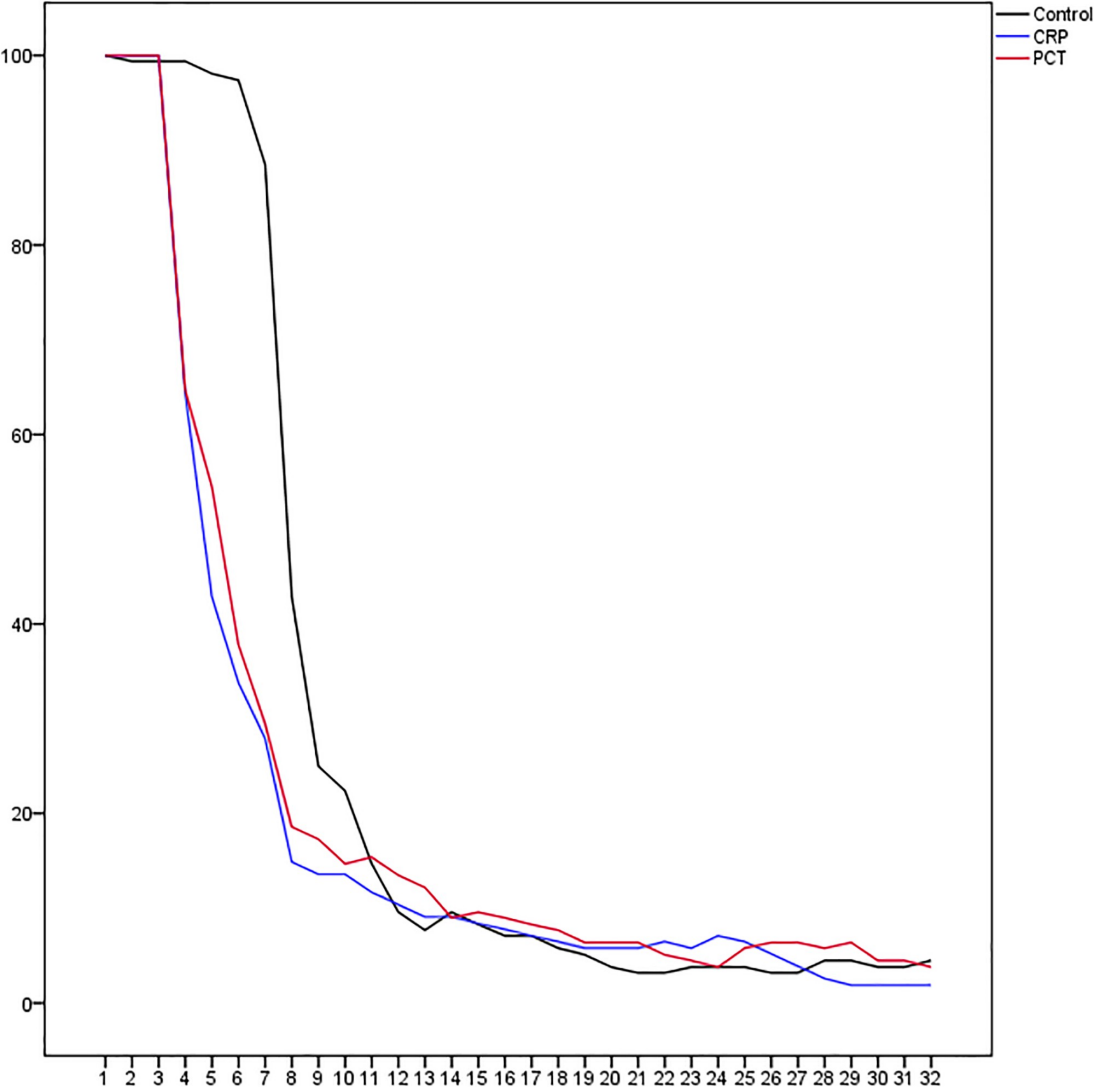
Percentage of patients on antibiotic treatment per day.

Overall antibiotic use was reduced in both intervention groups, respectively 30% in the CRP group (median 4 vs. 7d; p <0.001) and by 22% (median 5.5 vs. 7d; p<0.001) in the PCT group (Table 2). The rate of patients on antibiotic treatment during admission and follow-up is shown in Fig 2. Primary outcome data were incomplete for two patients in the CRP group, because full information of antibiotic use up to day 30 was missing. Both patients were, therefore, excluded from analysis. Sensitivity analyses (assuming both patients had either not or both had received additional antibiotics for seven days) yielded similar interpretation. All patients complied with the study algorithm and stopped antibiotics when so instructed. In a post-hoc analysis the rate ratio of receiving antibiotics during the first 30 days was 1.11 (95% CI 0.93–1.32; p = 0.129) for patients in the PCT compared to those in the CRP group.

**Table 2:** Primary outcome^a^.

	Control group, n = 156	CRP group, n = 154^b^	PCT group, n = 156
Days on antibiotic treatment			
Primary analysis			
Median (IQR)	7 (7–10)	4 (3–7)	5,5 (3–9)
RR (95% CI)	Reference	0.70 (0.61–0.82)	0.78 (0.68–0.89)
Sensitivity analysis 1: observed treatment days			
RR (95% CI)	Reference	0.73 (0.62–0.85)	0.78 (0.68–0.90)
Sensitivity analysis 2: observed treatment days +7			
RR (95% CI)	Reference	0.74 (0.63–0.86)	0.78 (0.68–0.90)

^a^ In the originally planned analyses using non-parametric tests all p values comparing the CRP and PCT group to the control group were below p<0.001

^b^ In the main analysis, two patients were excluded due to missing post-discharge antibiotic treatment data. In the sensitivity analyses all 156 patients were included

### Secondary outcomes

All secondary outcomes are summarized in Table 3. Additional antibiotics were prescribed in 82 (17.5%) patients. In 39 additional patients antibiotic treatment changed before day four and were not included in this analysis. A complete analysis including these patients appears in the S3 Table. In the intervention period a new antibiotic prescription was issued in 24 (15.4%), 23 (14.7%) and 35 (22.4%) patients in the control group, CRP group and PCT group respectively. The daily hazard ratios for new antibiotic prescriptions compared to standard care, were 0.99 (95% CI 0.56–1.76; p = 0.97) for the CRP group and 1.63 (95% CI 0.97–2.75; p = 0.064) for the PCT group. Reasons for new antibiotic prescriptions are listed in the S4 Table. Among those randomised to CRP-based treatment, a new course of antibiotics was started after discontinuation of the initial course based on the algorithm in 11 (7%) patients, and this occurred in 30 (19%) patients randomised to PCT-based treatment. Case summaries for all these patients can be found in the S3 Appendix.

**Table 3:** Secondary outcomes.

	Control group, n = 156	CRP group, n = 156	PCT group, n = 156
New antibiotic prescriptions			
Intervention period day 4-30(±2)			
Number of cases (%)	24 (15.4)	23 (14.7)	35 (22.4)
HR (95% CI)	Reference	0.99 (0.56–1.76)	1.63 (0.97–2.75)
		p = 0.97	p = 0.064
Time to clinical stability^a^			
Median days (IQR)	3 (1–5)	2 (1–4)	3 (2–5)
Number of cases (%)	142 (91)	138 (88.5)	138 (88.5)
HR (95% CI)	Reference	1.07 (0.85–1.36)	0.85 (0.67–1.08)
		p = 0.55	p = 0.19
Length of stay			
Median days (IQR)	4.5 (3–7)	4 (3–6)	5 (3–8)
Cox regression HR (95% CI)	Reference	0.93 (0.74–1.17)	0.82 (0.66–1.03)
Competing events regression model^b^		0.98 ((0.80–1.19)	0.84 ((0.69–1.02)
30- day mortality			
Number of cases (%)	2 (1.3)	2 (1.3)	5 (3.2)

^a^ In total 50/468 patients did not meet the criteria, 18 were in the CRP group, 18 in the PCT group and 14 in the control group. This was mainly due to either an elevated heart rate >100 bpm or low arterial oxygen saturation on room air that was due to known comorbidities (mainly COPD).

^b^ Analysed using a competing risk regression model with death as a competing variable.

9 patients (1.9%) had succumbed at day 30; 2 (1.3%) in the control group, 2 (1.3%) in the CRP group and 5 (3.2%) in the PCT group. Only 2 of these patients, both in the PCT group, received a shorter course of antibiotics according to study protocol. In both patients antibiotics were restarted due to relapsing fever. One patient died due to in-hospital aspiration on the day she was set to be discharged. The other patient was treated for three days according to PCT levels, discharged at day four and readmitted three days after discharge with recurring pneumonia from which he recovered and was discharged. Two weeks after discharge he died from euthanasia due to end-stage COPD. In all other patients (n = 5) that died before day 30 biomarkers remained high during antibiotic treatment, with the consequence that antibiotics could not be stopped before day seven.

## Discussion

In this randomised controlled trial both strategies of feedback of results from CRP-based and PCT-based algorithms for discontinuation of antibiotic treatment reduced antibiotic exposure during a 30 day follow-up period, compared to standard care in patients hospitalized with CAP in non-ICU wards.

Studies have shown that antibiotic duration can be shortened based on clinical parameters, such as the criteria for clinical stability defined in the IDSA guidelines [5, 7, 19]. However, some patients do not reach these criteria, even at the time of discharge. Furthermore, despite all evidence that shorter antibiotic courses are safe, most physicians still treat patients hospitalized with CAP for 7–10 days [20, 21]. Although Dutch guidelines have recommended antibiotic courses of 5 days for patients with CAP and good clinical recovery by day 3 since 2011, 18 of 156 (11.5%) patients in the standard care treatment group were treated for less than 7 days in the current study. In another more recently performed Dutch multi-centre study addressing a similar patient population the average duration of antibiotic treatment was 6.6 days [10]. Antibiotic use is an important driver of antimicrobial resistance, and shortening of treatment duration reduces antibiotic selective pressure. This underlines the need for simple and objective criteria to change clinical practice.

In our study both CRP-based and PCT-based algorithms reduced the median days of antibiotic use in the first 30 days after admission from 7 to 4 and 5.5, respectively. A CRP based algorithm has advantages over a PCT based algorithm. CRP is a widely used, cheap(er) and readily available biomarker in nearly every clinical setting. Point-of-care CRP testing has proven effective in reducing antibiotic consumption for lower respiratory tract infections in nursing homes [22].

Yet, there is little evidence to support CRP measurements to tailor the duration of antibiotic treatment in patients with CAP. In one study failure of CRP to decline within the first few days of hospitalization was associated with a poor prognosis of CAP [23]. Only once has a CRP based algorithm been compared to a PCT based algorithm [24]. In that trial of 94 ICU-patients with sepsis, 49 were allocated to PCT and 45 to CRP measurements, without a control group. Median duration of treatment was 6 days in the CRP group and 7 days in the PCT group, with no differences in outcomes between groups. The same group studied a modified version of their CRP algorithm in open label RCT in ICU patients and found a small reduction in antibiotic treatment time in favour of the CRP group [25]. However, their algorithm differs from ours and a reduction of 50% was needed for patients with an initial CRP >100 mg/L and an absolute value of <35 mg/L was needed for patients with an initial value <100 mg/L.

Our PCT-based algorithm reduced antibiotic exposure but resulted in slightly more new antibiotic prescriptions during follow-up compared to the control group. This might have resulted, in part, from the fact that less patients in the PCT group received empirical therapy that covered atypical pathogens than the other study groups (Table 1), which might have made them more prone to a change in antibiotic regime. Several other large, well-designed trials and a Cochrane review have been conducted in a variety of clinical settings but none of those reported more treatment failure in the PCT groups [11, 12].

Christ-Crain et al. showed that in patients with radiologically proven CAP a PCT based algorithm reduced the duration of antibiotic treatment to a median of 5 days with similar rates of antibiotic prescription in long term follow-up. However, their algorithm was different from ours, they gave clinicians more freedom to take their own judgement into account and their definition of treatment failure was limited to symptoms related to CAP. Lastly combination therapy was started in 34% of patients, as compared to 24% of patients in our study.

Despite all evidence addressing PCT, there are still concerns about exclusion rates in clinical trials, uncertainty with regards to the necessity of overruling of the PCT algorithm in trials by treating physicians and the usefulness of PCT in patients with atypical pathogens, COVID-19, renal failure or critically ill patients [13, 14, 26–28].

Our study has several limitations. First, our study was underpowered to exclude harm due to reduced duration of antibiotic treatment, as recently recommended by the Joint Programming Initiative on Antimicrobial Resistance (JPIAMR) working group on design of antimicrobial stewardship evaluations [29]. Ideally such a trial would include all patients admitted with CAP and should demonstrate superiority in reducing antibiotic exposure over standard clinical practice and simultaneously demonstrate non-inferiority design for unwanted clinical outcomes. Short antibiotic courses carry the risk of undertreatment leading to recurrence or worsening of symptoms, additional antibiotic prescriptions, and increased time to recovery. Based on a meta-analysis of 26 RCT’s there is no evidence that PCT based treatment strategies carry any of these risks [11]. In the current study 41 of 312 patients received a new antibiotic prescription after a short initial course based on the biomarker algorithms, and 23 of these prescriptions occurred within a week after antibiotic treatment was stopped. It is unlikely that all of these could have been prevented with a longer initial course, since some of these were due to culture results that returned resistant pathogens to empiric therapy.

The potential harm of short antibiotic treatment should be weighed against the harms of excess antibiotic treatment. Excess antibiotic treatment does not seem associated with lower rates of adverse outcomes, including death, readmission and emergency department visits. However, excess treatment duration is associated with higher rates of patient-reported adverse events and on a population level leads to more resistant pathogens due to higher selective antibiotic pressure. This underlines the importance of antibiotic stewardship not only for the population, but on the patient level as well [30].

Second, CRP measurements were frequently used in our control group and to a lesser extent in our PCT group. It is unclear if these measurements influenced clinical decision making and potentially study outcomes. If they did it would most likely lead to an underestimation of the effect of our CRP algorithm. In our PCT group CRP measurements did not influence antibiotic treatment duration. Even if they did, it would lead to an overestimation of the effect of the PCT algorithm.

Third, the observed 30-day mortality rate in our study is relatively low (1.9%), even though 15% of our patients classified as severe pneumonia according to the CURB-65 score. Reported 30-day mortality rates for hospitalised non-ICU patients with CAP range from 5–10%. The low mortality could have resulted from the study design, in which patients had to decide on day 2 or 3 on study participation, which may have selected for a less sicker study population. For instance informed consent on admission was not possible e.g. due to delirium in 53 patients, which could, therefore, not be included. Overall 450 out of 1434 screened patients were eligible for the intervention but were not included or randomized, compared to 468 randomized patients. This limits the generalisability of our findings. Fourth, post-discharge sampling to determine biomarkers was part of study protocol, but may not be realistic in routine daily care practices. In 62 of 88 patients in whom a blood sample was taken at home or on an outpatient visit, antibiotics were discontinued because of biomarker measurements. This also limits the generalisability of our results. Fifth, the feedback of biomarker results to treating physicians was an important part of the intervention tested, implying that the effectiveness of the intervention may well be less when implemented without active feedback. Lastly, there is a considerable publication delay. However, the research question regarding biomarker based strategies is still relevant today, and the average treatment durations in our control group are comparable to those reported in similar patient populations in recently published studies [7, 10].

In conclusion, in this study both CRP and PCT based treatment algorithms reduced the duration of antibiotic treatment in patients admitted to a regular hospital ward with CAP. Future studies should focus on the non-inferiority of this approach with respect to clinically relevant patient centered outcomes.

## Supporting information

S1 Appendix Complete in- and exclusion criteria. (DOCX)

S2 Appendix Criteria for clinical stability. (DOCX)

S3 Appendix Case summaries. (DOCX)

S1 Table Results of microbial tests. (DOCX)

S2 Table Overview of infections and co-infections. (DOCX)

S3 Table New antibiotic prescriptions from day 1 till 30±2. (DOCX)

S4 Table Reasons for new antibiotic prescriptions in the intervention period. (DOCX)

S1 Fig Overview of biomarker assessment per day in the control group. (DOCX)

S2 Fig Overview of biomarker assessment per day in the CRP group. (DOCX)

S3 Fig Overview of biomarker assessment per day in the PCT group. (DOCX)

S1 Checklist CONSORT 2010 checklist of information to include when reporting a randomised trial*. (DOC)

S1 Protocol (PDF)
