# Supplementary material for: Biomarker guided antibiotic stewardship in community acquired pneumonia: A randomized controlled trial
Source: PLoS One. 2024 Aug 20;19(8):e0307193. doi: 10.1371/journal.pone.0307193 (PMC11335096; doi:10.1371/journal.pone.0307193)
Supplement: S1 Appendix — (DOCX) [file pone.0307193.s001.docx]

**S1 File. Complete in- and exclusion criteria**

**Inclusion criteria**

- Age ≥18
- Estimated life expectancy > 30 days
- Initial admission to a non-ICU ward
- A new infiltrate on chest radiograph
- Presence of one or more of the following signs and symptoms:
  - Temperature ≥ 38⁰C
  - Dyspnoea
  - Cough (with or without expectoration of sputum)
  - Chest pain
  - Malaise or fatigue
  - Gastro-intestinal symptoms,
  - Rales/rhonchi or wheezing,
  - Egophony or bronchial breath sounds
  - Haemoptysis.

**Exclusion criteria**

- Severe immunosuppression as judged by the investigator (e.g. HIV infection, chemotherapy, immunosuppressive drugs with exclusion of low-dose corticosteroids)
- Active neoplastic disease
- Obstruction pneumonia
- Aspiration pneumonia
- Pneumonia that developed within eight days of hospital discharge
- Expected inability to comprehend or follow the study protocol
- Pregnancy
- Lactation
- Unable to give informed consent (either patient or legal representative)
- Suspected non-respiratory infection diagnosed prior to randomisation and requiring antibiotic treatment
