# Supplementary material for: Biomarker guided antibiotic stewardship in community acquired pneumonia: A randomized controlled trial
Source: PLoS One. 2024 Aug 20;19(8):e0307193. doi: 10.1371/journal.pone.0307193 (PMC11335096; doi:10.1371/journal.pone.0307193)
Supplement: S2 Appendix — (DOCX) [file pone.0307193.s002.docx]

**S2 Appendix. Criteria for clinical stability**

- Temperature ≤37.8°C
- Heart rate ≤100 beats/min
- Respiratory rate ≤24 breaths/min
- Systolic blood pressure ≥90 mm Hg
- Arterial oxygen saturation ≥90% or pO2 ≥60 mm Hg on room air
- Ability to maintain oral intake
- Normal mental status
