# Supplementary material for: Biomarker guided antibiotic stewardship in community acquired pneumonia: A randomized controlled trial
Source: PLoS One. 2024 Aug 20;19(8):e0307193. doi: 10.1371/journal.pone.0307193 (PMC11335096; doi:10.1371/journal.pone.0307193)
Supplement: S3 Appendix — (DOCX) [file pone.0307193.s003.docx]

**S3 Appendix. Case summaries for all patients with a new antibiotic prescription after a shorter initial course of antibiotics based on the biomarker algorithms**

n = 30 PCT group

n = 11 CRP group

5 – PCT group

Antibiotics stopped on admission day 4 after 3 days of treatment with amoxicillin (=admission day 4) based on PCT levels. Microbiology results yielded B. pertussis. Developed fever the next day (=admission day 5) and was treated for a hospital acquired pneumonia. CRP admission day one 201, PCT 0.08. CRP on admission day four 54, PCT 0.06 CRP on admission day five 83. Was treated with antibiotics for an additional 12 days.

19 – CRP group

Antibiotics stopped on admission day 5, after 4 days of treatment based on CRP levels. CRP on admission day one 75, on admission day five 26. Antibiotics were restarted on admission day 7 due to insufficient recovery with a sputum culture yielding a H. influenza. Patient received antibiotics for an additional 38 days.

26 – PCT group

Antibiotics stopped on admission day 5, after 4 days of treatment based on PCT levels. PCT on admission day one 1.59, on admission day five 0.15. On day 24 patient presented with an acute exacerbation of COPD with signs of a lower respiratory tract infection and received antibiotics for an additional 7 days.

32 – PCT group

Antibiotics stopped on admission day 5, after 4 days of treatment based on PCT levels. Patient had a persisting fever at that point, but his clinical condition and clinical parameters all improved. Antibiotics were restarted the next day due to fear of under treatment. PCT on admission day one was 0.22, on admission day five 0.05. Microbial tests only yielded a parainfluenzavirus. Patient received antibiotics for an additional 8 days.

34 – PCT group

Antibiotics stopped on admission day 5, after 4 days of treatment based on PCT levels. Antibiotics were restarted the next day due to purulent sputum, sub-febrile temperature and a CRP of 360. Sputum culture yielded S. pneumonia, M. Catarrhalis, H. parainfluenzae. CRP on admission day 1 was 336, PCT 10.30 On admission day five PCT was 0.11. Patient received antibiotics for an additional 7 days.

36 – PCT group

Antibiotics stopped on admission day 4, after 3 days of treatment based on PCT levels. Antibiotics were restarted on admission day 8 due to fever that was attributed to the CAP. Microbial tests yielded no pathogens. PCT on admission day one 0.05, CRP 1. On admission day 4 PCT 0.06, On admission day 5 CRP was 82. Patient received antibiotics for an additional 11 days.

53 – CRP group

Antibiotics stopped on day 4, after 3 days of treatment based on CRP levels. Microbial tests only yielded an influenzavirus. CRP on admission day one 50 on admission day four 14. Patient was discharged on admission day 7 and took additional antibiotics she had at home for 5 days due to complaints of increased cough and sputum volume. She was eventually readmitted 5 days later with sputum culture yielding a P. aeruginosa which the previous antibiotics did not cover, so she received additional antibiotic treatment for 20 days.

56 – CRP group

Antibiotics stopped on admission day 5, after 4 days of antibiotic treatment based on CRP levels. Antibiotics restarted 3 days later due to an increase in dyspnea, sputum volume with sputum culture yielding a P. Aeruginosa. CRP on admission day one 32, on admission day five 6. Patient received additional antibiotic treatment for 7 days.

58 – PCT group

Antibiotics stopped on admission day 6, after 5 days of treatment based on PCT levels. Microbial tests yielded S. pneumoniae(urine antigen test), enterobacter spp. (sputum culture) and rhinovirus (oralpharyngeal swab). PCT on admission day one 4.9, on admission day six 0.40. Patient had fully recovered clinically by that point and was discharged. He presented himself to our ER department 5 days later with chest pains. A pulmonary embolism was ruled out, CRP was 12 and he was discharged without additional therapy. He was then re-admitted 30 days after the initial admission with meningitis/encephalitis and received additional antibiotics for 4 days until he died.

65 – PCT group.

Antibiotics stopped on admission day 4, after 3 days of treatment based on PCT levels. On the outpatient visit on day 32 patient presented with signs of a lower respiratory tract infection and received additional antibiotics for 7 days.

85 – CRP group.

Antibiotics stopped on admission day 4, after 3 days of treatment based on CRP levels. Antibiotics were restarted on admission day 5 due to recurring fever. Microbial tests yielded no pathogens. CRP on admission day one 164, on admission day four 82, on admission day five 118. Antibiotics were restarted for 7 days, patient recovered and was discharged on admission day 14. He was then re-admitted a week later with an exacerbation of COPD due to a recurring pneumonia and antibiotics were restarted for another 7 days. In total he received additional antibiotics for 14 days.

95 – PCT group

Antibiotics stopped on admission day 4, after 3 days of treatment based on PCT levels. Microbial tests yielded no pathogens. Patient recovered and was discharged on admission day 4. Nine days later patient was readmitted with an aspiration pneumonia and treated for an additional 7 days.

132 – PCT group

Antibiotics stopped on admission day 6, after 5 days of treatment based on PCT levels. Patient recovered quickly and was discharged on admission day 5. A pathogen was never identified. PCT on admission day one 3.89 on admission day six 0.39. 17 days later patient was prescribed an additional 7 days of antibiotics due to an otitis media.

138 – PCT group

Antibiotics stopped on admission day 4, after 3 days of treatment based on PCT levels. Oropharyngeal PCR yielded M. pneumoniae. Patient was discharged on admission day 10. PCT on admission day one 0.10 on admission day four 0.05. He was readmitted 19 days after discharge due to a urinary tract infection associated with an indwelling catheter and received antibiotics for an additional 28 days.

141 – CRP group

Antibiotics stopped on admission day 6, after 5 days of treatment based on CRP levels. Microbial tests yielded a H. parainfluenzae. Patient was discharged on admission day 5. He was readmitted 13 days later with heart failure with possible signs of a lower respiratory tract infection and received additional antibiotics for 7 days.

169 – PCT group

Antibiotics stopped on admission day 4, after 3 days of treatment based on PCT levels. Microbial tests yielded a H. parainfluenzae, K. oxytoca and human metapneumovirus. PCT on admission day one 0.07 on admission day four 0.05. Patient was recovering slowly even after stopping antibiotics, but due to a resistant pathogen for the empiric antibiotic treatment and prolonged clinical recovery (dyspnea, need for supplemental oxygen and sputum retention) extra antibiotics were started on admission day 11 for an additional 7 days. Patient was eventually discharged on admission day 17.

179 – PCT group

Antibiotics stopped on admission day 4, after 3 days of treatment based on PCT levels. Microbial tests yielded a M. pneumoniae. PCT on admission day one 0.19 on admission day four 0.05. CRP on admission day one 130 on admission day five 79. Empiric treatment happened to cover mycoplasma spp in this patient. However, the day the antibiotics were discontinued, the PCR came back positive and antibiotics were restarted by the treating physician due to fear of possible undertreatment, despite a good clinical recovery at that point. He was discharged on admission day 8. Patient received additional antibiotics for 21 days.

192 – CRP group

Antibiotics stopped on admission day 4, after 3 days of treatment based on CRP levels. Microbial tests yielded H. Influenzae and rhinovirus. Patient was discharged on admission day 9. CRP on admission day one 97 on admission day four 13. Antibiotics were restarted on admission day 6 due to recurring signs of a lower respiratory tract infection. Patient received additional antibiotic therapy for 7 days.

212 – CRP group.

Antibiotics stopped on admission day 7, after 6 days of treatment based on CRP levels. Microbial tests yielded a S. Aureus (sputum culture) and a human metapneumovirus. CRP on admission day one 22 on admission day seven 9. Patient recovered after initial treatment and was discharged on admission day 7. She was then readmitted 15 days later with recurring symptoms indicative of a lower respiratory tract infection/pneumonia and received antibiotics for an additional 7 days.

213 – CRP group

Antibiotics stopped on admission day 4, after 3 days of treatment based on CRP levels. Microbial tests yielded no pathogens. CRP on admission day one was 54 CRP on admission day four 19. Patient recovered quickly and was discharged on admission day 5. Patient was readmitted 8 days later due to an exacerbation of COPD with signs of a lower respiratory tract infection and received antibiotics for an additional 5 days.

220 – PCT group

Antibiotics stopped on admission day 4, after 3 days of treatment based on PCT levels. Microbial tests yielded no pathogens. PCT on admission day one 0.21 on admission day four 0.23. Patient was discharged on admission day 11. Patient was recovering slowly, but due to a persisting fever antibiotics were restarted on admission day 8 also covering atypical pathogens.

221 – PCT group

Antibiotics stopped on admission day 6, after 5 days of treatment based on PCT levels. Microbial tests yielded respiratory syncytial virus. PCT on admission day one 4.01 on admission day six 0.20. Patient was discharged on admission day seven. 18 days later he was readmitted due to recurring pneumonia and received an additional 7 days of antibiotic treatment.

232 – PCT group

Antibiotics stopped on admission day 6, after 5 days of treatment based on PCT levels. Microbial tests yielded S. pneumoniae, E. Coli and parainfluenzavirus. PCT on admission day one 0.64 on admission day six 0.11. Patient was discharged on day 6. On the outpatient visit on day 28 his chest x-ray barely improved and he had signs of a lower respiratory tract infection/pneumonia. He received an additional 7 days of antibiotics.

242 – PCT group

Antibiotics stopped on admission day 6 after 5 days of treatment based on PCT levels. Microbial tests yielded K. pneumoniae. PCT on admission day one 4.92 on admission day six 0.25. Patient was discharged on admission day 7. Patient was readmitted 14 days later with a septic shock, was admitted to the ICU and received additional antibiotics for 15 days.

250 – PCT group

Antibiotics stopped on admission day 7 after 6 days of treatment based on PCT levels. Microbial tests yielded S. pneumoniae. PCT on admission day one 3.62 on admission day seven 0.22. Patient was discharged on admission day 6. Patient was readmitted with a non-infectious exacerbation of COPD 10 days later and received no antibiotics, recovered and was discharged 5 days later. 4 days later (25 days after the initial admission) she presented to the ER department with clinical signs of recurring pneumonia, was admitted and treated for a hospital acquired pneumonia. She received additional antibiotic therapy for 7 days.

260 – PCT group

Antibiotics stopped on admission day 5 after 4 days of treatment based on PCT levels. Microbial tests yielded S. pneumoniae, S. Aureus (sputum culture) and rhinovirus. The same day antibiotics were restarted in the evening shift due to concerns of under treatment. The next morning antibiotics were again discontinued by the treating physician. Patient received 1 extra day of antibiotics.

279 – PCT group

Antibiotics stopped on admission day 4 after 3 days of treatment based on PCT levels. Microbial tests yielded no pathogens. PCT on admission day one 0.13 on admission day four 0.08.

Antibiotics were restarted 2 days later due to recurring fever. Patient received an additional 6 days of antibiotics.

320 – PCT group

Antibiotics stopped on admission day 4 after 3 days of treatment based on PCT levels. Microbial tests yielded no pathogens. PCT on admission day one 0.10 on admission day four 0.10.

On admission day 9 antibiotics were restarted due to recurring signs of a lower respiratory tract infection. Patient received an additional 14 days of antibiotic treatment.

331 – PCT group

Antibiotics stopped on admission day 4 after 3 days of treatment based on PCT levels. Microbial tests yielded H. Influenzae. PCT on admission day one 0.13 on admission day four 0.13. CRP on admission day one 197, on admission day five 134. Antibiotics were restarted the same day due to persistent fever and fear of an atypical pathogen. Patient received an additional 9 days of antibiotics.

348 – PCT group

Antibiotics stopped on admission day 4 after 3 days of treatment based on PCT levels. Microbial tests yielded rhinovirus. PCT on admission day one 0.19 on admission day four 0.12. CRP on admission day one 194 and on admission day five 174. Antibiotics were restarted on admission day 5 due to persistent fever. Despite the fever and slowly declining biomarker levels all other symptoms improved and patient was set to be discharged on admission day 6. On the day of her discharge she was found dead in her room due to aspiration of food leading to asphyxia. Patient received an additional day of antibiotics.

378 – PCT group

Antibiotics stopped on admission day 4 after 3 days of treatment based on PCT levels. Microbial tests yielded M. Catarrhalis and rhinovirus. PCT on admission day one 0.73 on admission day four 0.20. Patient visited his general practitioner 17 days after antibiotics were discontinued with complaints of fever and increased sputum volume. Patient received an additional 7 days of antibiotics.

395 – CRP group

Antibiotics stopped on admission day 5, after 4 days of treatment based on CRP levels. Microbial tests yielded S. pneumoniae and influenzavirus. CRP on admission day one was 130 CRP on admission day five 43. After stopping of antibiotic treatment patient had a recurring fever on the same day and received an additional 6 days of antibiotic treatment.

396 – PCT group

Antibiotics stopped on admission day 4 after 3 days of treatment based on PCT levels. Microbial tests yielded H. Influenzae, S. Aureus (sputum culture) and M. pneumoniae. PCT on admission day one 0.12 on admission day four 0.05. Patient was discharged on admission day 5 and presented himself to the ER department 6 days later complaining of dyspnea but no other symptoms and received an additional 7 days of antibiotic treatment targeting the M. pneumoniae.

399 – CRP group

Antibiotics stopped on admission day 4, after 3 days of treatment based on CRP levels. Microbial tests yielded S. pneumoniae and human metapneumovirus. CRP on admission day one was 236 CRP on admission day four 85. During the outpatient visit on day 32 patient presented with signs of a lower respiratory tract infection and received an additional 7 days of antibiotic treatment.

403 – PCT group

Antibiotics stopped on admission day 4 after 3 days of treatment based on PCT levels. Microbial tests yielded H. Influenzae. PCT on admission day one 0.19 on admission day four 0.11. Patient was recovering and clinical signs of pneumonia were improving, but remained oxygen dependent for some time. On admission day 11 she had a worsening hypoxia and a recurring fever and was treated for a Hospital-acquired pneumonia. She received 10 days of additional antibiotic treatment.

405 – PCT group

Antibiotics stopped on admission day 4 after 3 days of treatment based on PCT levels. Microbial tests yielded S. Aureus (sputum culture) and influenzavirus. PCT on admission day one 0.87 on admission day four 0.15. Patient recovered and was discharged on admission day 6. Ten days after discharge patient phoned the outpatient clinic complaining of dyspnea and fatigue. The S. Aureus in the sputum culture was resistant for the empiric antibiotic coverage she received earlier. Patient received an additional 7 days of antibiotic treatment targeting the S. Aureus.

409 – PCT group

Antibiotics stopped on admission day 5 after 4 days of treatment based on PCT levels. Microbial tests yielded S. pneumoniae and influenzavirus. PCT on admission day one 0.97 on admission day four 0.11. Patient was discharged on admission day 5. Two days after discharge she presented to another hospital with persisting dyspnea and was readmitted. She received an additional 7 days of antibiotic treatment.

415 – PCT group

Antibiotics stopped on admission day 4 after 3 days of treatment based on PCT levels. Microbial tests yielded S. pneumoniae, H. parainfluenzae and influenzavirus. PCT on admission day one 0.24 on admission day four 0.05. Patient was recovering and was discharged on admission day 4, but had a recurring fever prior to discharge. The treating physician chose to continue antibiotic treatment for another 6 days.

416 – CRP group

Antibiotics stopped on admission day 4 after 3 days of treatment based on CRP levels. Microbial tests yielded no pathogens. CRP on admission day one 77 PCT 0.08 on admission day four CRP was 29. Patient was discharged on admission day 4. Sixteen days after discharge patient had recurring signs of a lower respiratory tract infection and received an additional 7 days of antibiotic treatment.

432 – PCT group

Antibiotics stopped on admission day 4 after 3 days of treatment based on PCT levels. Microbial tests yielded M. pneumoniae. PCT on admission day one 0.09 on admission day four 0.06. Patient was recovering well with empiric antibiotic treatment that did not cover the M. pneumoniae. When the M. pneumoniae was found the treating physician decided to treat the patient for another 15 days specifically targeting the M. pneumoniae due to fear of under treatment. Patient was discharged on admission day 4.

444 – PCT group

Antibiotics stopped on admission day 4 after 3 days of treatment based on PCT levels. Microbial tests yielded H. Influenzae and E. Coli. PCT on admission day one 0.21 on admission day four 0.19. Patient recovered and was discharged on admission day 4. Three days later patient had recurring signs of a lower respiratory tract infection, was readmitted and treated for another 6 days for a hospital acquired pneumonia. He died due to unknown causes on day 23, eleven days after completion of his treatment for the hospital acquired pneumonia.
