# Supplementary material for: Biomarker guided antibiotic stewardship in community acquired pneumonia: A randomized controlled trial
Source: PLoS One. 2024 Aug 20;19(8):e0307193. doi: 10.1371/journal.pone.0307193 (PMC11335096; doi:10.1371/journal.pone.0307193)
Supplement: S1 Table — (DOCX) [file pone.0307193.s004.docx]

**S1 Table. Results of microbial tests^a^**

|  | Sputum culture (n=297) | Blood culture (n=372) | UAT (pneumococci n=419, L. pneumophila n=415) | Oropharyngeal swab (PCR, n=436) | Any test  n, (% of all participants) |
| --- | --- | --- | --- | --- | --- |
| *S. pneumoniae* | 42 | 36 | 64 |  | 112 (23.9) |
| *L. pneumophila* | 5 |  | 8 |  | 9 (1.9) |
| *M. catarrhalis* | 23 |  |  |  | 23 (4.9) |
| *H. influenzae* | 45 | 2 |  |  | 47 (10.0) |
| *H. parainfluenzae* | 37 |  |  |  | 37 (7.9) |
| *S. aureus* | 19 |  |  |  | 19 (4.1) |
| *P. aeruginosa* | 8 |  |  |  | 8 (1.7) |
| *K. pneumoniae* | 9 |  |  |  | 9 (1.9) |
| *K. oxytoca* | 3 |  |  |  | 3 (0.6) |
| *E. coli* | 16 | 1 |  |  | 16 (3.4) |
| *S. marcescens* | 3 |  |  |  | 3 (0.6) |
| Other Enterobacteriaciae | 8 |  |  |  | 8 (1.7) |
| Other gram negatives | 16 |  |  |  | 16 (3.4) |
| Contaminants |  | 18 |  |  | 18 (3.8) |
|  |  |  |  |  |  |
| *M. pneumoniae* |  |  |  | 23 | 23 (4.9) |
| *C. pneumoniae* |  |  |  | 1 | 1 (0.2) |
| *B. pertussis* |  |  |  | 2 | 2 (0.4) |
| Adenovirus |  |  |  | 1 | 1 (0.2) |
| Rhinovirus |  |  |  | 55 | 55 (11.7) |
| Influenzavirus |  |  |  | 38 | 38 (8.1) |
| Parainfluenzavirus |  |  |  | 11 | 11 (2.3) |
| hMPV |  |  |  | 15 | 15 (3.2) |
| RSV |  |  |  | 12 | 12 (2.6) |
| Coronavirus |  |  |  | 10 | 10 (2.1) |
| Total | 235 | 57 | 72 | 168 | 496 |

^a^ UAT = Urinary Antigen Test, hMPV = Human Metapneumovirus, RSV = Respiratory Syncytial Virus
