# Supplementary material for: Biomarker guided antibiotic stewardship in community acquired pneumonia: A randomized controlled trial
Source: PLoS One. 2024 Aug 20;19(8):e0307193. doi: 10.1371/journal.pone.0307193 (PMC11335096; doi:10.1371/journal.pone.0307193)
Supplement: S2 Table — (DOCX) [file pone.0307193.s005.docx]

**S2 Table. Overview of infections and co-infections**

|  | N (% of total patients) |
| --- | --- |
| 1 bacterial pathogen | 117(25) |
| 2 bacterial pathogens | 31 (6.6) |
| 3 bacterial pathogens | 8 (1.7) |
| 4 bacterial pathogens | 0 (0) |
| 5 bacterial pathogens | 1 (0.2) |
| 1 viral pathogen | 55 (11.7) |
| 2 viral pathogens | 1 (0.2) |
| 1 bacterial and 1 viral pathogen | 49 (10.5) |
| 2 bacterial and 1 viral pathogen | 27 (5.8) |
| 3 bacterial and 1 viral pathogen | 4 (0.8) |
| 4 bacterial and 1 viral pathogen | 3 (0.6) |
| 1 bacterial and 2 viral pathogen | 1 (0.2) |
| Total | 297 (63.5) |
