# Supplementary material for: Biomarker guided antibiotic stewardship in community acquired pneumonia: A randomized controlled trial
Source: PLoS One. 2024 Aug 20;19(8):e0307193. doi: 10.1371/journal.pone.0307193 (PMC11335096; doi:10.1371/journal.pone.0307193)
Supplement: S3 Table — (DOCX) [file pone.0307193.s006.docx]

**S3 Table. New antibiotic prescriptions from day 1 till 30±2**

|  | Control group, n=156 | CRP group, n=156 | PCT group, n=156 |
| --- | --- | --- | --- |
| New antibiotic prescriptions |  |  |  |
| Number of cases (%)  HR (95% CI) | 34 (21.8)  Reference | 35 (22.4)  1.05 (0.66 - 1.68)  p = 0.84 | 52 (33.3)  1.65 (1.07 - 2.55)  p = 0.023 |
