# Supplementary material for: Biomarker guided antibiotic stewardship in community acquired pneumonia: A randomized controlled trial
Source: PLoS One. 2024 Aug 20;19(8):e0307193. doi: 10.1371/journal.pone.0307193 (PMC11335096; doi:10.1371/journal.pone.0307193)
Supplement: S4 Table — (DOCX) [file pone.0307193.s007.docx]

**S4 Table. Reasons for new antibiotic prescriptions in the intervention period**

|  | Control, n (% of group) | PCT, n (% of group) | CRP, n (% of group) | Total, n (% of all patients) |
| --- | --- | --- | --- | --- |
| Recurring fever/new infection | 10 (6.4) | 21 (13.5) | 12 (7.7) | 43 (9.2) |
| Persisting fever | 0 (0) | 4 (2.6) | 3 (1.9) | 7 (1.5) |
| Empyema | 8 (5.1) | 0 (0) | 1 (0.6) | 9 (1.9) |
| Fear of under treatment despite good clinical recovery | 3 (1.9) | 3 (1.9) | 1 (0.6) | 7 (1.5) |
| Insufficient clinical recovery | 1 (0.6) | 2 (1.3) | 2 (1.3) | 5 (1.1) |
| Other infection | 1 (0.6) | 3 (1.9) | 1 (0.6) | 5 (1.1) |
| Pathogen resistant to empiric therapy | 1 (0.6) | 2 (1.3) | 2 (1.3) | 5 (1.1) |
| Cryptogenic Organizing Pneumonia | 0 (0) | 0 (0) | 1 (0.6) | 1 (0.2) |
| Total n (%) | 34 (21.8) | 52 (33.3) | 35 (22.4) | 121 (25.8) |
