# Supplementary figures and images for: Biomarker guided antibiotic stewardship in community acquired pneumonia: A randomized controlled trial
Source: PLoS One. 2024 Aug 20;19(8):e0307193. doi: 10.1371/journal.pone.0307193 (PMC11335096; doi:10.1371/journal.pone.0307193)

**S1 Fig. Overview of biomarker assessment per day in the control group**


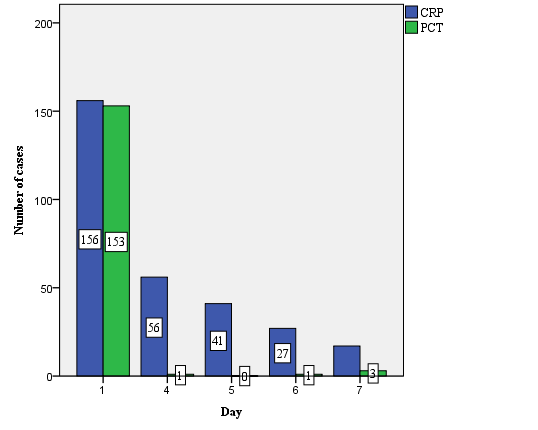

Supplement: S1 Fig — (DOCX) [file pone.0307193.s008.docx]

**S2 Fig. Overview of biomarker assessment per day in the CRP group**


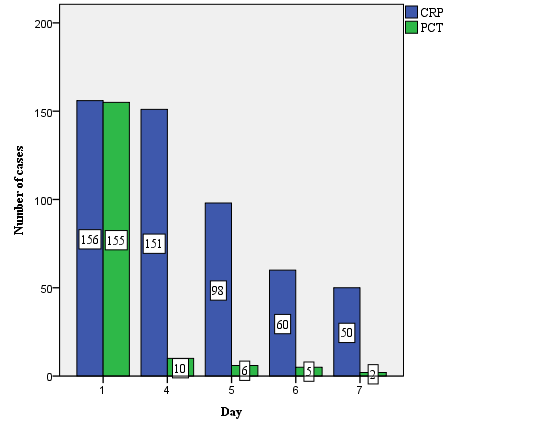

Supplement: S2 Fig — (DOCX) [file pone.0307193.s009.docx]

**S3 Fig. Overview of biomarker assessment per day in the PCT group**


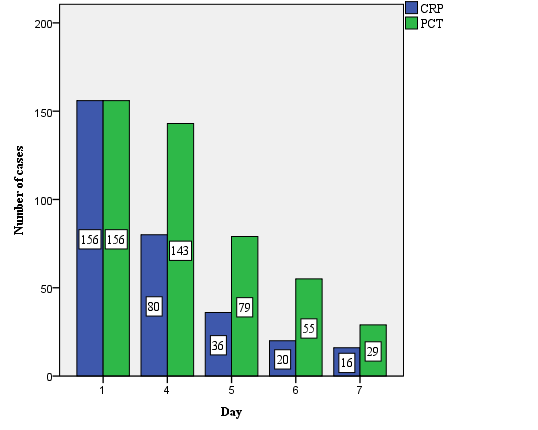

Supplement: S3 Fig — (DOCX) [file pone.0307193.s010.docx]
