# Supplementary material for: Biomarker guided antibiotic stewardship in community acquired pneumonia: A randomized controlled trial
Source: PLoS One. 2024 Aug 20;19(8):e0307193. doi: 10.1371/journal.pone.0307193 (PMC11335096; doi:10.1371/journal.pone.0307193)
Supplement: S1 Protocol — (PDF) [file pone.0307193.s012.pdf]

INDEX

1. Introduction 3
2. Objectives 5
    2.1 Primary objective(s) 5
    2.2 Secondary objective(s) 5
3. Study design 5
4. Study population 5
    4.1 Population 6
    4.2 Inclusion criteria 6
    4.3 Exclusion criteria 6
    4.4 Sample size calculation 6
5. Treatment of subjects 7
    5.1 Days 1,2 and 3 – all groups 7
    5.2 Days 4 and after – common clinical practice group 7
    5.3 Days 4 and after – PCT guided group 7
    5.4 Days 4 and after – CRP guided group 7
6. Methods 7
    6.1 Study parameters 7
        6.1.1 Main study parameter 7
        6.1.2 Secondary study parameters 7
    6.2 Randomisation and treatment allocation 8
    6.3 Study procedures 8
    6.4 Study procedures – detailed overview 8
        6.4.1 Day 1 – hospital admission. 8
        6.4.2 Days 2 and 3 9
        6.4.3 Days 4-7 9
        6.4.4 Day 14 10
        6.4.5 Day 30 (outpatient visit) 10
    6.5 Study evaluations 10
        6.5.1 Definitions of clinical response 10
        6.5.2 Bacteriological evaluation 10
        6.5.3 Radiological evaluation 11
        6.5.4 Laboratory evaluation 11
    6.6 Withdrawal of individual subjects 11
    6.7 Follow-up of subjects withdrawn from treatment 11
7. Safety reporting and monitoring 11
    7.1 Section 10 WMO event 11
    7.2 Adverse events 12
    7.3 Serious adverse events 12
    7.4 Follow-up of adverse events 12
    7.5 Data Safety Monitoring Board 12
8. Data analysis 12
    8.1 Calculation of study numbers 12
    8.2 Randomisation method 13
    8.3 Analysis populations 13
        8.3.1 Clinical Intent-To-Treat (ITT) group 13
        8.3.2 Per protocol analysis 13
    8.4 Methods of analysis 14
        8.4.1 General statistical considerations 14
        8.4.2 Comparison of baseline characteristics 14
        8.4.3 Efficacy analyses 14
        8.4.4 Clinical response analysis 14
    8.5 Methodology 14
9. Ethical considerations 15
    9.1 Regulation statement 15

9.2 Recruitment and consent 15
9.3 Benefits and risks assessment, group relatedness 15
9.4 Compensation for injury 15
10. Administrative aspects, monitoring and publication 15
10.1 Handling and storage of data and documents 15
10.2 Monitoring and quality assurance 16
10.3 Amendments 16
10.4 Annual progress report 16
10.5 End of study report 16

1. Introduction

Community-acquired pneumonia (CAP) is a common and serious illness. In developed countries it is the most important cause of death due to an infectious disease and in the Netherlands it is the fourth leading cause of death overall. [1-3]

In CAP caused by bacteria prompt initiation of antibiotic therapy is recommended, since a delay might be associated with increased mortality.[4;5] The optimal duration of antibiotic therapy in bacterial CAP remains unknown.[6] Most likely, it varies from patient to patient.

Current guidelines recommend treatment duration of 7-21 days, depending on illness severity and type of pathogen.[2;7-9] However, adherence to guidelines is variable and physicians tend to treat longer, especially in patients with comorbidities and patients with severe CAP.[10;11] Duration of treatment can be guided by clinical signs, but interpretation of the clinical response lacks standardization and is prone to interobserver variability.[12]

A new approach to estimate the presence of an infection and response to treatment is the use of biomarkers.[13;14] Circulating levels of calcitonin precursors, including procalcitonin are elevated in bacterial infections.[15;16] Procalcitonin can follow either a classic hormonal expression pathway or in the presence of an infection, a cytokine-like expression pathway.[16;17]

The release of procalcitonin during infection can be induced either directly by microbial toxins such as endotoxin and indirectly by humoral factors such as IL-1β, TNF-α and IL-6 or the cell-mediated host response. [16;17]

Several studies have shown procalcitonin can be used as a marker for bacterial lower respiratory infections and CAP. [18-20] Some studies demonstrate procalcitonin can be used effectively and safely as a marker to initiate or discontinue treatment with antibiotics.[11;21-23] However only one of these trials focuses on patients with CAP admitted to hospital. One of our objectives is to validate the procalcitonin based treatment strategy mentioned by Christ Crain et al. and Long et al. [23;24]

A different way to assess the presence of (bacterial) infection is measuring the blood level of C-Reactive Protein (CRP). An elevated CRP correlates only with (systemic) inflammation and not per se infection. Several studies have attempted to use CRP as a marker in lower respiratory tract infections and CAP in a primary care setting. [25-29] Over the years several reviews have questioned the use of CRP in patients with community acquired pneumonia/lower respiratory tract infections as a marker to initiate or withhold antibiotic treatment. [27;30] There is however evidence that supports the use of consecutive measurements of CRP in follow up of antibiotic treatment in CAP. Several studies showed that a delayed normalisation of CRP within the first 3–7 days of follow-up is suggestive of inappropriate antibiotic therapy and eventually treatment failure. [31-34]

Using the database from the CAPISCE study[35] we retrospectively derived a CRP based treatment strategy which we believe can be used just as effectively as the PCT based strategy to discontinue antibiotic treatment in patients with CAP admitted to hospital.

Our main goal is to determine whether the CRP and PCT guided strategies can be used to safely and effectively reduce the duration of antibiotic treatment in patients with CAP admitted to hospital. The rationale and criteria of the two different strategies is outlined in chapter 4.4

2. Objectives

2.1 Primary objective(s)
The primary objective of this study is to determine whether a CRP guided and PCT guided treatment strategy (strategies mentioned below) can be used to safely and effectively reduce the duration of antibiotic treatment as compared to common clinical practice in patients with CAP requiring hospitalisation.

The diagnosis CAP will be made if a non-hospitalized person presents with one or more symptoms associated with a lower respiratory tract infection **and** a new infiltrate on the chest radiograph. These symptoms are: temperature greater than 38°C (100.4°F); cough with or without sputum; hemoptysis; pleuritic chest pain; dyspnea; malaise or fatigue; myalgia; gastro-intestinal symptoms; rales, rhonchi or wheezing; egophony or bronchial breath sounds.

The 3 different treatment strategies are as follows:
1. Treatment according to common practice: often a 7 day course of antibiotics.
2. Treatment with antibiotics for at least 3 days according to current guidelines, with extension of antibiotic treatment according to procalcitonin levels. Antibiotic treatment will be discontinued if the procalcitonin level is below 0.25 mcg/L **or** shows a reduction to 10% of the initial value.
3. Treatment for 3 days according to current guidelines, with extension of antibiotic treatment according to CRP levels. Antibiotic treatment will be discontinued if the value is below 100 mg/L **and** shows a reduction to 50% of the initial value.

2.2 Secondary objective(s)
The secondary objectives of this study are to assess the length of hospital stay, clinical response, 30-day mortality, time to clinical stability and relapse rate within 30 days. A relapse is defined as new or worsening symptoms indicative of pneumonia after initial improvement on therapy. Furthermore several biomarkers and genetic polymorfisms for corticosteroid-receptors will be assessed at the end of the study.

3. Study design
This study will be set up as a Randomised Controlled Trial with a parallel design, patients will be randomly allocated to one of 3 treatment groups. No blinding will be performed, since treating physicians will have to make a decision to continue or withhold treatment based on laboratory evaluations. In order to make the right decision they will have to know whether or not the patient is receiving antibiotics.
Patients will be included at hospital admission and the follow up period is 1 month. The control group will consist of patients getting treated according to common clinical practice.
Patients will be admitted to hospital and receive therapy according to the study protocol. They will be discharged from the hospital when their medical condition and social situation is stable. If patients are discharged and still using antibiotics, blood tests will be performed daily in the participating centre and the researcher will contact the patients by phone to inform them whether or not antibiotic treatment can be discontinued. After discharge the patient will be evaluated at an outpatient visit on day 30. In case of any event an interim visit will be scheduled. If for some reason a patient fails to perform an outpatient blood test, antibiotics will be continued until the next blood test or for a total of 7 days and the reason for failure will be documented.
This study will be conducted in the Medical Centre Alkmaar, the Isala clinics in Zwolle and the Slotervaart hospital in Amsterdam.

4. Study population

4.1 Population

A total of 468 patients will be enrolled into this study and will be divided into three equally large groups.

4.2 Inclusion criteria

Male and female patients with a diagnosis of CAP and all criteria listed below:

1. Age 18 or above, no upper age limit will be employed.
2. Patients must require hospitalisation.
3. Clinical presentation of an acute illness with one or more of the following symptoms:
   a. Temperature ≥ 38.0 ^{0}C (100.4°F)
   b. Dyspnoea
   c. Cough (with or without expectoration of sputum)
   d. Chest pain
   e. Malaise or fatigue
   f. Myalgia
   g. Gastro-intestinal symptoms
   h. Rales, rhonchi or wheezing
   i. Egophony or bronchial breath sounds
   j. Hemoptysis.
4. New consolidation(s) on the chest radiograph.
5. Written informed consent obtained.
6. (Pre-event) Life expectancy > 30 days.

4.3 Exclusion criteria

Subjects presenting with any of the following will not be included in the study:

1. Severe immunosuppression (HIV infection, chemotherapy).
2. Active neoplastic disease.
3. Obstruction pneumonia (e.g. from lung cancer).
4. Aspiration pneumonia.
5. Pneumonia that developed within 8 days after hospital discharge.
6. Unable and/or unlikely to comprehend and/or follow the protocol.
7. Pregnant and/or lactating women.

4.4 Sample size calculation.

The objective of this study is to assess the efficacy of two different treatment regimens as compared to current general practice. The primary analysis will be the comparison of the duration of antibiotic treatment of the CRP-guided treatment group compared to common practice and of the PCT-guided treatment group compared to common practice in the intent-to-treat (ITT) population. A per-protocol analysis will be performed as well.
We used the criteria mentioned above for CRP and PCT (procalcitonin level below 0.25 mcg/L **or** a reduction to 10% of the initial value; CRP below 100 mg/L **and** a reduction to 50% of the initial value) to retrospectively perform an analysis on the database from the CAPISCE trial[35] to predict a mean and a standard deviation of antibiotic treatment for both groups in order to perform the power analysis. We then retrospectively selected 170 cases from the CAP-START trial to derive a mean and standard deviation for the common clinical practice group. The results of all groups are listed below:

**Duration of theoretical antibiotic treatment in the CRP guided group**					
	N	Minimum	Maximum	Mean	Std. Deviation
crpdag	196	1,00	14,00	5,1327	3,61518

Duration of theoretical antibiotic treatment in the PCT guided group					
	N	Minimum	Maximum	Mean	Std. Deviation
pctdag	173	1,00	14,00	6,2832	4,80257

Duration of antibiotic treatment in the Common Clinical Practice group					
	N	Minimum	Maximum	Mean	Std. Deviation
pctdag	170	0,00	42,00	8,7588	5,87499

We assume a mean difference of 2 days of antibiotic treatment to be clinically relevant. The results will be evaluated for a normal distribution using the kolmogorov-smirnov test. If they are normally distributed an ANOVA will be performed, with the addition of a Tukey-HSD test. If they are not normally distributed a Kruskall-Wallis test will be performed with the addition of separate Mann-Whitney U tests as post-hoc tests. The significance of these separate Mann-Whitney U tests will be corrected for multiple testing using the Bonferroni Holm method.
Using the means and standard deviations mentioned above, with an alpha of 0.025 (corrected for multiple testing using the Bonferroni Holm method) and a β of 0.20 this would require a total of 139 patients per group if the results are normally distributed and 146 patients per group if they aren't.
A total of 148 patients per group will be included, to account for loss to follow-up, deaths etc. Which amounts to a total of 468 patients. Calculation was done by using the program G-power.

5. Treatment of subjects

5.1 Days 1,2 and 3 – all groups
All patients will receive treatment with broad-spectrum antibiotics for the first 3 days, which may be switched to small-spectrum antibiotics if a causative agent is found. This is in line with current guidelines.

5.2 Days 4 and after – common clinical practice group
The patients in the common clinical practice group will receive antibiotics for a total of 7 days if no complications occur. Total treatment duration may be lengthened by the treating physician. The researcher will document the reason for this choice when this occurs.

5.3 Days 4 and after – PCT guided group
Each day a blood test will be performed assessing the PCT level in the blood. If this shows a procalcitonin level below 0.25 mcg/L **or** a reduction to 10% of the initial value treatment will be discontinued. After the decision to discontinue treatment is made no further blood tests will be performed unless the patient has worsening symptoms.

5.4 Days 4 and after – CRP guided group
Each day a blood test will be performed assessing the CRP level in the blood. If this shows a CRP below 100 mg/L **and** a reduction to 50% of the initial value treatment will be discontinued. After the decision to discontinue treatment is made no further blood tests will be performed unless the patient has worsening symptoms.

6. Methods

6.1 Study parameters
6.1.1 Main study parameter

The primary objective of this study is to determine whether the CRP and PCT strategies can be used to safely and effectively reduce the duration of antibiotic treatment in patients with CAP requiring hospitalisation.

6.1.2 Secondary study parameters

The secondary objectives of this study are to assess the length of hospital stay, clinical response, 30-day mortality, time to clinical stability and relapse rate within 30 days. A relapse is defined as new or worsening symptoms indicative of pneumonia after initial improvement on therapy. Furthermore several biomarkers and genetic polymorfisms for corticosteroid-receptors will be assessed at the end of the study.

6.2 Randomisation and treatment allocation

Randomisation will be done by using block-randomisation with a block size of 30 patients, which amounts to a total of 15 blocks of 30 patients and 1 block of 18 patients. The allocation ratio in each block will be 1:1:1. So each block will contain 10 patients getting treated according to Common Clinical Practice, 10 patients getting treated according to the CRP guided strategy and 10 patients getting treated according to the PCT guided strategy. The last block will contain 6 patients in each treatment group.
Each centre will start with a single block, upon completion they will be assigned the next available block. For example if centre A starts with block 1 and centre B starts with block 2, the centre who includes the first 30 patients will be assigned block 3. The main-investigator will be responsible for maintaining this list and appointing blocks to each centre.
No stratification will be performed, since there are no known or expected variables to warrant stratification.
Randomisation will be done by using a validated internet based randomisation strategy which can be found at sealedenvelope.com.
Once a number is assigned to a patient, the investigator will open the treatment regimen assignment and the subject will receive treatment according to the assigned strategy.

6.3 Study procedures

All diagnostic and other procedures are outlined in appendix C, a distinction is made between common clinical practice and extra procedures patients will undergo if they choose to participate in this study.

Subjects will be evaluated for clinical stability every day of hospitalization. Laboratory evaluations will be performed for hospitalised patients at day 1 and days 3-7 until the threshold level is reached. Any laboratory evaluation after day 7 will be on indication. Microbiological evaluations will be determined at baseline and if applicable on day 30. An outpatient visit will be scheduled at day 30. A detailed overview of the different procedures is outlined below.

6.4 Study procedures – detailed overview.

6.4.1 Day 1 – hospital admission.

At the baseline visit (hospital admission, day 1), subjects will be deemed eligible to take part in the study after the clinical diagnosis of Community Acquired Pneumonia has been made. The following procedures will be completed:

1. Verify inclusion/exclusion criteria
2. Obtain written informed consent
3. Record medical history
4. Collection of demographic information, concurrent disease, concomitant medication use and previous antibiotic therapy.

5. Physical examination (including but not limited to: mild/moderate/severe illness, sputum characteristics, cough, dyspnoea, pleuritic chest pain, chills or rigors and lung sounds) and vital signs (blood pressure, heart rate, temperature, respiration rate and oxygen saturation) will be recorded.
6. The CURB-65 score will be determined. (**C**onfusion, **U**rea> 7 mmol/l, **R**espiratory rate ≥30/min, diastolic **B**lood pressure ≤ 60 en age ≥ **65** years)
7. Blood and urine sample for routine laboratory testing and inflammation study will be obtained.
8. Chest X-rays will be made, if possible both PA and lateral views will be obtained. An AP chest X-ray will be considered acceptable if the patient is unable to stand.
9. Blood cultures will be obtained for all. All blood cultures will be assessed by the laboratory of medical microbiology. All isolates will be tested for susceptibility.
10. If possible an adequate sputum sample will be collected for conventional microbiologic assessment as well as PCR assays and delivered expeditiously to the laboratory to ensure a viable specimen. Freshly expectorated purulent sputum samples must be examined macroscopically for consistency and colour and microscopically to determine the suitability for culture. The presence of > 25 polymorphonuclear leucocytes and < 10 squamous epithelial cells per low power field of a Gram stained specimen is defined as "representative" sputum. Only "representative" sputum specimens as determined by Gram stain should be cultured. If medically indicated, the investigator may attempt to obtain other lower respiratory secretions such as bronchial washing and Broncho Alveolar Lavage (BAL) obtained by bronchoscopy. The specimens will be handled in the same way as sputum specimens.
11. Urine will be collected for detection of *S. pneumoniae* urinary antigen and for *Legionella pneumophila* (subtype 1) urinary antigen.
12. A nasopharyngeal swab will be taken and will be stored for possible future research.
13. Extra blood sample(s) will be taken to assess genetic polymorphisms of corticosteroid-receptors and several other biomarkers.
14. If pleural fluid is present on the Chest X-ray and deemed clinically relevant, a thoracocenthesis will be performed. Pleural fluid will be handled in the same way as sputum specimens.
15. Patients will be randomised into 1 of three possible treatment strategies.
16. Treatment according to current guidelines will be administered.

6.4.2 Days 2 and 3

1. Vital signs (pulse, respiration rate, heart rate, blood pressure, and body temperature, Oxygen Saturation) will be recorded three times a day. Each day time to clinical stability will be assessed.
2. Laboratory examination: on indication.
3. Clinical improvement/failure is scored according to Chow et al[36] (appendix B). If treatment needs to be changed because the subjects fails to improve clinically then the subject should be evaluated as a treatment failure and appropriate therapy instituted. This therapy should be recorded.
4. If possible patients will be switched from iv therapy to oral therapy. It is appropriate to switch to oral medication in the following situations: [5;9]
    a. Hemodynamically stable
    b. If respiratory symptoms such as cough, fever, shortness of breath, and chest pain are improving.
    c. Oral intake and gastrointestinal absorption adequate.

6.4.3 Days 4-7

1. Vital signs (pulse, respiration rate, heart rate, blood pressure, and body temperature, Oxygen Saturation) will be recorded three times a day if the patient is still admitted to hospital.

2. Laboratory examination: CRP, PCT, WBC and on indication other tests will be performed until the patient is discharged.
3. Clinical improvement/failure is scored according to Chow et al[36] (appendix B). If treatment needs to be changed because the subjects fails to improve clinically then the subject should be evaluated as a treatment failure and appropriate therapy instituted. This therapy should be recorded.
4. If possible patients will be switched from iv therapy to oral therapy. It is appropriate to switch to oral medication in the following situations:
    a. Oral temperature or equivalent <37.8 ^{o}C (100.3 ^{o}F) for at least 24 hours.
    b. If respiratory symptoms such as cough, shortness of breath, and chest pain are improving.
    c. Oral intake and gastrointestinal absorption adequate.
    d. White blood cell count normalizing.
5. Patients may be discharged from the hospital if they meet the following criteria [9]:
    a. No longer require iv medication, criteria for switching from iv to oral medication mentioned above.
    b. If their clinical and social situation is stable.
        i. Clinical stability is defined as:
            1. Temperature <37.8 ^{o}C (100.3 ^{o}F)
            2. Heart rate <100 beats/min
            3. Respiratory rate < 24 breaths/minute
            4. Systolic blood pressure > 90 mm Hg
            5. Arterial oxygen saturation >90% or pO2 >60 mm Hg on room air
            6. Ability to maintain oral intake
            7. Normal mental status
6. Treatment may be discontinued in the CRP group if the patient shows clinical improvement and the CRP is below 100 mg/L **and** shows a reduction to 50% of the initial value.
7. Treatment may be discontinued in the PCT group if the patient shows clinical improvement and the PCT level is below 0.25 mcg/L **or** shows a reduction to 10% of the initial value.

6.4.5 Day 30 (outpatient visit)

1. Signs and symptoms identified during the previous days should be assessed. In addition, any new signs and symptoms should be reported.
2. If applicable a sputum sample will be obtained.
3. A chest X-ray will be performed to evaluate radiological response.
4. A pulmonary function test with diffusion capacity will be performed.
5. Investigator evaluation of clinical response will be recorded.
6. On indication laboratory examination may be performed.

6.5 Study evaluations

6.5.1 Definitions of clinical response [5;9]

The overall clinical response will be determined by the investigator on day 30. Clinical response will be classified by the investigator as cure, failure, or indeterminate at day 30 as defined in Appendix B. When the investigator is unsure on how to score selected cases, he will consult a colleague to try and reach a consensus. Clinical evaluation of response will be based on the global assessment of the clinical presentation of the subject and compared to the baseline assessment. Clinical assessment will be based upon resolution or improvement of radiological and clinical signs of infection such as resolution of fever, disappearance or diminution in purulent sputum production, and improvement or resolution of dyspnoea, cough and leucocytosis, as well as improvement in general physical condition.

6.5.2 Bacteriological evaluation

Routine culture techniques and susceptibility testing of isolates should be conducted in the laboratory as appropriate. The results of antigen testing will be interpreted according to the manufacturer's information.

6.5.3 Radiological evaluation
Consecutive chest X-rays will be evaluated and their evolution will be graded as:
Resolution: disappearance of all radiological signs of infection.
Improvement: significant improvement in the radiological signs of infection compared to baseline.
Failure: no change or worsening in the radiological signs of infection compared to baseline.

6.5.4 Laboratory evaluation
All clinically significant abnormal laboratory tests occurring during the study will be repeated at appropriate intervals until they either return to baseline or to a level deemed acceptable by the investigator/treating physician.

6.6 Withdrawal of individual subjects
Subjects can leave the study at any time for any reason if they wish to do so without any consequences. The investigator or treating physician can decide to withdraw a subject from the study for urgent medical reasons. If possible the researcher will document the reason for withdrawal.

6.7 Follow-up of subjects withdrawn from treatment
Where possible all patients that cease to participate in the study will be followed up according to the schedule of the study protocol. If they refuse, they will return to their own treating physician.

7. Safety reporting and monitoring

7.1 Section 10 WMO event
In accordance to section 10, subsection 1, of the WMO, the investigator will inform the subjects and the reviewing accredited METC if anything occurs, on the basis of which it appears that the disadvantages of participation may be significantly greater than was foreseen in the research proposal. The study will be suspended pending further review by the accredited METC, except insofar as suspension would jeopardise the subjects' health. The investigator will take care that all subjects are kept informed.

7.2 Adverse events
Events involving adverse drug reactions, illnesses with onset during the study, or exacerbations of pre-existing illnesses should be recorded. Exacerbation of pre-existing illness, including the disease under study, is defined as a manifestation (sign or symptom) of the illness that indicates a significant increase in the severity of the illness as compared to the severity noted at the start of the trial. It may include worsening or increase in severity of signs or symptoms of the illness, an increase in frequency of signs and symptoms of an intermittent illness, or the appearance of a new manifestation/complication. Exacerbation of a pre-existing illness should be considered when a patient/subject requires new or additional concomitant drug or non-drug therapy for the treatment of that illness during the trial. Lack of or insufficient clinical response, benefit, efficacy, therapeutic effect, or pharmacological action, should not be recorded as an adverse event. The investigator must make the distinction between exacerbation of pre-existing illness due to a complication or manifestation unrelated to the causative agent being treated and lack of therapeutic efficacy.
All adverse events will be reported every 6 months by the investigator through the webportal "ToetsingOnline."

7.3 Serious adverse events

A serious adverse event is any untoward medical occurrence or effect that at any dose:

- Results in death
- Is life threatening at the time of the event
- Requires hospitalisation or prolongation of existing inpatients' hospitalisation
- Results in persistent or significant disability or incapacity

Any serious adverse event will be reported to the sponsor as soon as possible. This will be done by phone or email. In all centres the responsibility for reporting lies with the investigator coordinating the trial in the respective centre.
The sponsor will report the SAEs through the webportal "ToetsingOnline" to the accredited METC that approved the protocol, within 24 hours after the sponsor has first knowledge of the serious adverse event.

7.4 Follow-up of adverse events

All AEs will be followed until they have ablated, or until a stable situation has been reached. Depending on the event, follow up may require additional tests or medical procedures as indicated, and/or referral to the general physician or a medical specialist. SAEs need to be reported till end of study within the Netherlands, as defined in the protocol.

7.5 Data Safety Monitoring Board

The DSMB consists of 3 members who are independent of the set-up and execution of the trial. At least one DSMB member will be an independent statistician. Another member with knowledge about the disease should be represented. The third member should have knowledge about safety effects and ethical dilemmas. The statistician is also the chairman of the board. All members of the DSMB receive the definite protocol of the present study and all revisions. The statistician of the DSMB will convene every six to twelve months and review the results, i.e. results split by experimental and control arms. Only if necessary, the other members of the board will be informed about the interim results. However, the DSMB has the power to recommend termination of the study based on the evaluation of these results. There are typically three reasons a DSMB might recommend termination of the study: safety concerns, outstanding benefit, and futility. The primary mandate of the DSMB is to protect patient safety. If adverse events of a particularly serious type are more common in the experimental arm compared to the control arm, then the DSMB would have to strongly consider termination of the study. This evaluation has to be made in consideration of risk/benefit. In many cases, the experimental arm could cause serious adverse events , but the resulting improvement in survival could outweigh these adverse events.
At the conclusion of a DSMB meeting, the DSMB should inform the findings or conclusions to the study investigators by mail or e-mail. If there is any dispute between the DSMB and study investigators, the DSMB should notify the METC Noord Holland.

The DSMB consists of the following members:

R. Glandorf, biostatistician.
J.H. Ruiter, cardiologist.
E. van Melle, ethicist.

8. Data analysis

8.1 Calculation of study numbers

The objective of this study is to assess the efficacy of two different treatment regimens as compared to current general practice. The primary analysis will be the comparison of the duration of antibiotic

treatment of the CRP-guided treatment group compared to common practice and of the PCT-guided treatment group compared to common practice in the intent-to-treat (ITT) population. A per-protocol analysis will be performed as well.
We used the criteria mentioned above for CRP and PCT (procalcitonin level below 0.25 mcg/L **or** a reduction to 10% of the initial value; CRP below 100 mg/L **and** a reduction to 50% of the initial value) to retrospectively perform an analysis on the database from the CAPISCE trial[35] to predict a mean and a standard deviation of antibiotic treatment for both groups in order to perform the power analysis. We then retrospectively selected 170 cases from the CAP-START trial to derive a mean and standard deviation for the common clinical practice group. The results of all groups are listed below:

**Duration of theoretical antibiotic treatment in the CRP guided group**					
	N	Minimum	Maximum	Mean	Std. Deviation
crpdag	196	1,00	14,00	5,1327	3,61518

**Duration of theoretical antibiotic treatment in the PCT guided group**					
	N	Minimum	Maximum	Mean	Std. Deviation
pctdag	173	1,00	14,00	6,2832	4,80257

**Duration of antibiotic treatment in the Common Clinical Practice group**					
	N	Minimum	Maximum	Mean	Std. Deviation
dag	170	0,00	42,00	8,7588	5,87499

We assume a mean difference of 2 days of antibiotic treatment to be clinically relevant. The results will be evaluated for a normal distribution using the kolmogorov-smirnov test. If they are normally distributed an ANOVA will be performed, with the addition of a Tukey-HSD test. If they are not normally distributed a Kruskall-Wallis test will be performed with the addition of separate Mann-Whitney U tests as post-hoc tests. The significance of these separate Mann-Whitney U tests will be corrected for multiple testing using the Bonferroni Holm method.
Using the means and standard deviations mentioned above, with an alpha of 0.025 (corrected for multiple testing using the Bonferroni Holm method) and a β of 0.20 this would require a total of 139 patients per group if the results are normally distributed and 146 patients per group if they aren't.
A total of 156 patients per group will be included, to account for loss to follow-up, deaths etc. Which amounts to a total of 468 patients. Calculation was done by using the program G-power.

8.2 Randomisation method
Randomisation will be done by using block-randomisation with a block size of 30 patients, which amounts to a total of 15 blocks of 30 patients and 1 block of 18 patients. The allocation ratio in each block will be 1:1:1. So each block will contain 10 patients getting treated according to Common Clinical Practice, 10 patients getting treated according to the CRP guided strategy and 10 patients getting treated according to the PCT guided strategy. The last block will contain 6 patients in each treatment group.
Each centre will start with a single block, upon completion they will be assigned the next available block. For example if centre A starts with block 1 and centre B starts with block 2, the centre who includes the first 30 patients will be assigned block 3. The main-investigator will be responsible for maintaining this list and appointing blocks to each centre.
No stratification will be performed, since there are no known or expected variables to warrant stratification.

8.3 Analysis populations

8.3.1 Clinical Intent-To-Treat (ITT) group

All treated patients with clinical and radiological findings consistent with CAP will be included in the final ITT-population.

8.3.2 Per protocol analysis
All patients who were diagnosed with CAP and received treatment according to study protocol will be included in the per protocol analysis.
Patients will be considered as non-evaluable if they fulfil the following criteria:

1. Study medication discontinued earlier than planned or less than 80% of study drugs taken.
2. Received concomitant systemic antibiotics for intercurrent illnesses.
3. No visit at evaluation point, unless subject was previously deemed a treatment failure.
4. Patients admitted to ICU
5. Death within the first 3 days of treatment.

8.4 Methods of analysis

8.4.1 General statistical considerations
All data will be collected and analysed using SPSS statistics 20.
Statistical analysis will be performed on an intent-to-treat basis by an independent statistician. All continuous variables will be tested for normal distribution with the Kolmogorov-Smirnov test for normal distribution. The Student-t test will be used for normally distributed variables, the Mann-Whitney U test for not normally distributed variables and the χ^2 test for dichotomous variables. The software package SPSS 20 for Windows (IBM-SPSS Inc., Chicago, Illinois, USA) is available for statistical analysis. A value of $p<0.05$ will be considered significant.

All data will be collected and analysed using SPSS statistics 20.
Clinical response will be analysed by using the Kruskal wallis test.
Duration of treatment and in-hospital stay will be expressed as means with a 95% confidence interval. Sub-group analyses will be performed on patients with severe CAP according to the CURB criteria and patients with non-severe CAP.

8.4.2 Comparison of baseline characteristics
Baseline comparison of the treatment groups with regard to demographic variables (such as age, gender, number of co-morbidities, smoking, alcohol use, etc.) and severity of the disease.
Severity of disease will be assessed using the CURB-65 score.

8.4.3 Efficacy analyses
The primary efficacy analysis will be duration of antibiotic treatment.
The secondary efficacy analyses will be clinical response on day 30, length of hospital stay, time to clinical stability, relapse rate and overall mortality on day 30 and day 90.
Possible confounders (e.g. co-morbidities, type of pathogen etc.) will be assessed. Where appropriate a correction will be performed.

8.4.4 Clinical response analysis
Clinical response to therapy will be assessed by the investigator at day 30. Clinical response will be based on the global assessment of the clinical presentation of the subject made by the investigator at the evaluation time point compared to the baseline assessment. Clinical outcome by the end of treatment will be cure, improvement, or failure. A clinical outcome of “cure” or “improvement” is considered a success. At the visit on day 30, the clinical outcome will be defined as successful, failure or relapse according to criteria specified in Appendix B.
"Intent to treat" and "evaluable" clinical analyses will be performed comparing clinical outcomes as defined on day 30. Ninety-five percent (95%) confidence intervals on the difference between the treatment groups in the rates of clinical success will be computed.

8.5 Methodology

Statistical analysis will be performed on an intent-to-treat basis by the investigator and will be checked by an independent statistician. All continuous variables will be tested for normal distribution with the Kolmogorov-Smirnov test for normal distribution. The Student-t test will be used for normally distributed variables, the Mann-Whitney U test for not normally distributed variables and the χ^2 test for dichotomous variables. The software package SPSS 20 for Windows (IBM-SPSS Inc., Chicago, Illinois, USA) is available for statistical analysis. A value of $p<0.05$ will be considered significant.

9. Ethical considerations

9.1 Regulation statement

This study will be conducted in compliance with Good Clinical Practice (GCP), including the International Conference on Harmonization (ICH) guidelines and the most recent version of the Declaration of Helsinki.
All amendments to the protocol and informed consent forms must be reviewed by the local Independent Ethics Committee for approval before being implemented. In the event of any deviation from the protocol, the investigator must document the nature of and rationale for the deviation.
Informed consent must take place prior to any study specific procedure or test. Written signed and dated informed consent will be obtained from each subject (or his/her legally acceptable representative) in accordance with GCP and with local regulatory and legal requirements. The completed informed consent will be retained by the investigator as part of the study records.

9.2 Recruitment and consent

Patients or their legal representatives will be asked to participate in this study by the attending physician or the investigator at the time of hospital admission. All patients will receive the patient information letter and informed consent form. They will be given one hour to consider their decision. Prior to consent blood tests may be performed and antibiotics can be started, since this is all part of regular care and in the patients interest.

9.3 Benefits and risks assessment, group relatedness

Low risk study according to the risk-classification as designed by the Dutch Foundation of University Medical Centres. The full risk classification (in Dutch) can be found in Appendix A.

9.4 Compensation for injury

The sponsor/investigator has a liability insurance which is in accordance with article 7, subsection 6 of the WMO.

The sponsor (also) has an insurance which is in accordance with the legal requirements in the Netherlands (Article 7 WMO and the Measure regarding Compulsory Insurance for Clinical Research in Humans of 23th June 2003). This insurance provides cover for damage to research subjects through injury or death caused by the study.

1. € 450.000,-- (i.e. four hundred and fifty thousand Euro) for death or injury for each subject who participates in the Research;
2. € 3.500.000,-- (i.e. three million five hundred thousand Euro) for death or injury for all subjects who participate in the Research;
3. € 5.000.000,-- (i.e. five million Euro) for the total damage incurred by the organisation for all damage disclosed by scientific research for the Sponsor as 'verrichter' in the meaning of said Act in each year of insurance coverage.

The insurance applies to the damage that becomes apparent during the study or within 4 years after the end of the study.

10. Administrative aspects, monitoring and publication

10.1 Handling and storage of data and documents

All data will be collected by the researcher on the Case Report Form (CRF). The data will be entered into a database, where subjects will be assigned a study number. Their personal information will not be entered into the database. A separate subject identification list will be kept by the sponsor in case the study data needs to be linked to individual subjects.
All study data will be kept for 15 years. Blood samples and sputum samples will be stored for 3 years, to conduct future research regarding inflammatory biomarkers and genetic polymorphisms of corticosteroid receptors.

10.2 Monitoring and quality assurance

The conduct of the study and data entries will be monitored by an internal monitor. This will be done by H.J. Prins who will select a sample of cases and check them for irregularities. If any are found, the monitor may choose to select a larger sample or review all available data. He will report the irregularities to the sponsor who is responsible for correcting any mistakes.
All adverse events will be analysed periodically by the statistician of the DSMB. If adverse events of a particularly serious type are more common in the experimental arm compared to the control arm he will inform the sponsor and other members of the DSMB which may then take actions as described in chapter 7.5.
All serious adverse events will be reported to the accredited METC.

10.3 Amendments

Amendments are changes made to the research after a favourable opinion by the accredited METC has been given. Non-substantial amendments will not be notified to the accredited METC and the competent authority, but will be recorded and filed by the sponsor. All other amendments will be notified to the METC that gave a favourable opinion.

10.4 Annual progress report

The sponsor/investigator will submit a summary of the progress of the trial to the accredited METC once a year. Information will be provided on the date of inclusion of the first subject, numbers of subjects included and numbers of subjects that have completed the trial, serious adverse events, other problems, and amendments.

10.5 End of study report

The investigator will notify the accredited METC of the end of the study within a period of 8 weeks. The end of the study is defined as the last patient's last visit. In case the study is ended prematurely, the investigator will notify the accredited METC within 15 days, including the reasons for the premature termination. Within one year after the end of the study, the investigator/sponsor will submit a final study report with the results of the study, including any publications/abstracts of the study, to the accredited METC.

Reference List

1. Oosterheert JJ, Bonten MJ, Hak E, Lammers JW, Schneider MM, Hoepelman IM. [The increase in pneumonia-related morbidity and mortality among adults in the Netherlands and possible explanations for it]. *Ned Tijdschr Geneeskd* 2004;**148**:1765-9.

2. Niederman MS, Mandell LA, Anzueto A, Bass JB, Broughton WA, Campbell GD *et al*. Guidelines for the management of adults with community-acquired pneumonia. Diagnosis, assessment of severity, antimicrobial therapy, and prevention. *Am J Respir Crit Care Med* 2001;**163**:1730-54.

3. Mortensen EM, Coley CM, Singer DE, Marrie TJ, Obrosky DS, Kapoor WN *et al*. Causes of death for patients with community-acquired pneumonia: results from the Pneumonia Patient Outcomes Research Team cohort study. *Arch Intern Med* 2002;**162**:1059-64.

4. Meehan TP, Fine MJ, Krumholz HM, Scinto JD, Galusha DH, Mockalis JT *et al*. Quality of care, process, and outcomes in elderly patients with pneumonia. *JAMA* 1997;**278**:2080-4.

5. Wiersinga WJ, Bonten MJ, Boersma WG, Jonkers RE, Aleva RM, Kullberg BJ *et al*. SWAB/NVALT (Dutch Working Party on Antibiotic Policy and Dutch Association of Chest Physicians) guidelines on the management of community-acquired pneumonia in adults. *Neth.J Med* 2012;**70**:90-101.

6. File TM, Jr.,.Mandell LA. What is optimal antimicrobial therapy for bacteremic pneumococcal pneumonia? *Clin Infect Dis* 2003;**36**:396-8.

7. File TM, Jr. Clinical efficacy of newer agents in short-duration therapy for community-acquired pneumonia. *Clin Infect Dis* 2004;**39 Suppl 3**:S159-S164.

8. Mandell LA, Bartlett JG, Dowell SF, File TM, Jr., Musher DM, Whitney C. Update of practice guidelines for the management of community-acquired pneumonia in immunocompetent adults. *Clin Infect Dis* 2003;**37**:1405-33.

9. Mandell LA, Wunderink RG, Anzueto A, Bartlett JG, Campbell GD, Dean NC *et al*. Infectious Diseases Society of America/American Thoracic Society consensus guidelines on the management of community-acquired pneumonia in adults. *Clin Infect Dis* 2007;**44 Suppl 2**:S27-S72.

10. Mandell LA,.File TM, Jr. Short-course treatment of community-acquired pneumonia. *Clin Infect Dis* 2003;**37**:761-3.

11. Christ-Crain M, Jaccard-Stolz D, Bingisser R, Gencay MM, Huber PR, Tamm M *et al*. Effect of procalcitonin-guided treatment on antibiotic use and outcome in lower respiratory tract infections: cluster-randomised, single-blinded intervention trial. *Lancet* 2004;**363**:600-7.

12. Wipf JE, Lipsky BA, Hirschmann JV, Boyko EJ, Takasugi J, Peugeot RL *et al*. Diagnosing pneumonia by physical examination: relevant or relic? *Arch Intern Med* 1999;**159**:1082-7.

13. Muller B. Procalcitonin and ventilator-associated pneumonia: yet another breath of fresh air. *Am J Respir Crit Care Med* 2005;**171**:2-3.

14. Christ-Crain M,.Muller B. Procalcitonin in bacterial infections--hype, hope, more or less? *Swiss Med Wkly* 2005;**135**:451-60.

15. Muller B, Becker KL, Schachinger H, Rickenbacher PR, Huber PR, Zimmerli W *et al*. Calcitonin precursors are reliable markers of sepsis in a medical intensive care unit. *Crit Care Med* 2000;**28**:977-83.

16. Becker KL, Nylen ES, White JC, Muller B, Snider RH, Jr. Clinical review 167: Procalcitonin and the calcitonin gene family of peptides in inflammation, infection, and sepsis: a journey from calcitonin back to its precursors. *J Clin Endocrinol Metab* 2004;**89**:1512-25.

17. Linscheid P, Seboek D, Schaer DJ, Zulewski H, Keller U, Muller B. Expression and secretion of procalcitonin and calcitonin gene-related peptide by adherent monocytes and by macrophage-activated adipocytes. *Crit Care Med* 2004;**32**:1715-21.

18. Yealy DM,.Fine MJ. Measurement of serum procalcitonin: a step closer to tailored care for respiratory infections? *JAMA* 2009;**302**:1115-6.

19. Muller F, Christ-Crain M, Bregenzer T, Krause M, Zimmerli W, Mueller B *et al*. Procalcitonin levels predict bacteremia in patients with community-acquired pneumonia: a prospective cohort trial. *Chest* 2010;**138**:121-9.

20. Schuetz P, Christ-Crain M, Muller B. Procalcitonin and other biomarkers to improve assessment and antibiotic stewardship in infections--hope for hype? *Swiss Med Wkly* 2009;**139**:318-26.

21. Briel M, Schuetz P, Mueller B, Young J, Schild U, Nusbaumer C *et al*. Procalcitonin-guided antibiotic use vs a standard approach for acute respiratory tract infections in primary care. *Arch Intern Med* 2008;**168**:2000-7.

22. Schuetz P, Christ-Crain M, Thomann R, Falconnier C, Wolbers M, Widmer I *et al*. Effect of procalcitonin-based guidelines vs standard guidelines on antibiotic use in lower respiratory tract infections: the ProHOSP randomized controlled trial. *JAMA* 2009;**302**:1059-66.

23. Christ-Crain M, Stolz D, Bingisser R, Muller C, Miedinger D, Huber PR *et al*. Procalcitonin guidance of antibiotic therapy in community-acquired pneumonia: a randomized trial. *Am J Respir Crit Care Med* 2006;**174**:84-93.

24. Long W, Deng XQ, Tang JG, Xie J, Zhang YC, Zhang Y *et al*. [The value of serum procalcitonin in treatment of community acquired pneumonia in outpatient]. *Zhonghua Nei Ke Za Zhi* 2009;**48**:216-9.

25. Cals JW, Schot MJ, de Jong SA, Dinant GJ, Hopstaken RM. Point-of-care C-reactive protein testing and antibiotic prescribing for respiratory tract infections: a randomized controlled trial. *Ann Fam Med* 2010;**8**:124-33.

26. van dM, V, Neven AK, van den Broek PJ, Assendelft WJ. Diagnostic value of C reactive protein in infections of the lower respiratory tract: systematic review. *BMJ* 2005;**331**:26.

27. Christ-Crain M,.Opal SM. Clinical review: the role of biomarkers in the diagnosis and management of community-acquired pneumonia. *Crit Care* 2010;**14**:203.

28. Holm A, Nexoe J, Bistrup LA, Pedersen SS, Obel N, Nielsen LP *et al*. Aetiology and prediction of pneumonia in lower respiratory tract infection in primary care. *Br J Gen.Pract* 2007;**57**:547-54.

29. Holm A, Pedersen SS, Nexoe J, Obel N, Nielsen LP, Koldkjaer O *et al*. Procalcitonin versus C-reactive protein for predicting pneumonia in adults with lower respiratory tract infection in primary care. *Br J Gen.Pract* 2007;**57**:555-60.

30. Engel MF, Paling FP, Hoepelman AI, van dM, V, Oosterheert JJ. Evaluating the evidence for the implementation of C-reactive protein measurement in adult patients with suspected lower respiratory tract infection in primary care: a systematic review. *Fam Pract* 2012;**29**:383-93.

31. Bruns AH, Oosterheert JJ, Hak E, Hoepelman AI. Usefulness of consecutive C-reactive protein measurements in follow-up of severe community-acquired pneumonia. *Eur Respir J* 2008;**32**:726-32.

32. Coelho L, Povoa P, Almeida E, Fernandes A, Mealha R, Moreira P *et al*. Usefulness of C-reactive protein in monitoring the severe community-acquired pneumonia clinical course. *Crit Care* 2007;**11**:R92.

33. Menendez R, Cavalcanti M, Reyes S, Mensa J, Martinez R, Marcos MA *et al*. Markers of treatment failure in hospitalised community acquired pneumonia. *Thorax* 2008;**63**:447-52.

34. Smith RP, Lipworth BJ, Cree IA, Spiers EM, Winter JH. C-reactive protein. A clinical marker in community-acquired pneumonia. *Chest* 1995;**108**:1288-91.

35. Snijders D, Daniels JM, de Graaff CS, van der Werf TS, Boersma WG. Efficacy of corticosteroids in community-acquired pneumonia: a randomized double-blinded clinical trial. *Am J Respir Crit Care Med* 2010;**181**:975-82.

36. Chow AW, Hall CB, Klein JO, Kammer RB, Meyer RD, Remington JS. Evaluation of new anti-infective drugs for the treatment of respiratory tract infections. Infectious Diseases Society of America and the Food and Drug Administration. *Clin Infect Dis* 1992;**15 Suppl 1**:S62-S88.

Appendix A: Risico-classificatie ten behoeve van monitoring van investigator initiated onderzoek

**Toegevoegd risico**	
Wat is het toegevoegde risico van de onderzoekshandelingen voor de veiligheid van de proefpersoon?	Er zal enkele malen vaker bloed afgenomen worden in vergelijking met de reguliere klinische zorg.<br>Daarnaast zal de duur van de antibiotische therapie in 2 groepen verkort worden als de bloedwaarden dit toelaten. Mochten patiënten toch weer zieker worden zal dit hervat worden.

**Frequentie/schade**	
Hoe groot is de kans op schade?	klein
	Toelichting:<br>De complicaties van een vena punctie betreffen voornamelijk pijn, eventueel nabloeden en hematoom vorming. Dit is vrijwel altijd self-limiting en behoeft over het algemeen geen aanvullende therapie.<br><br>Het risico van een verkorte behandeling met antibiotica is dat patiënten na het staken van de therapie mogelijk weer klachten krijgen. De therapie zal niet gestaakt worden als de behandelend arts dit onveilig acht.<br>Over het algemeen geld dat het staken in een gecontroleerde omgeving gebeurt en de behandeling direct weer hervat kan worden als de behandelend arts dit besluit. Met patiënten in de thuissituatie zal worden afgesproken dat ze altijd direct contact op kunnen nemen met de dienstdoende assistent.

**Risico onderzoekshandeling**	
Hoeveelheid kennis en ervaring met de interventie, het geneesmiddel, voedingsmiddel of medisch hulpmiddel bij mensen?	Een venapunctie is een routine medische handeling waar veel ervaring mee is.<br>Er zijn reeds studies gedaan met het staken van antibiotica op geleide van het procalcitonine, dit bleek veilig en effectief.<br>Retrospectief onderzoek laat criteria voor de CRP groep zien die theoretisch net zo veilig en effectief zijn.
Bekende risico's:	Hematoom, nabloeding, pijn.<br>Bij de antibiotica groepen: theoretisch meer ziekte verschijnselen na vroeger staken.
Kans op het optreden van onbekende risico's:	Zeer klein.
Ernst van de mogelijke nadelige effecten/mate van schade:	☐ lichte schade
	Toelichting:<br>Hematoom, nabloeding en pijn zijn over het algemeen self-limiting zonder aanvullende therapie of diagnostiek.<br>Indien patiënten zieker worden na staken van antibiotica, zullen zij hun kuur mogelijk weer moeten hervatten zoals in de reguliere praktijk ook gebeurt.
Voorspelbaarheid van nadelig effect:	Direct merkbaar.
Mogelijkheid om ongewenste effecten te beheersen:	Bij nabloeding is afdrukken over het algemeen voldoende.<br>Bij zieker worden zal de antibiotica herstart worden.

Reversibiliteit van de mogelijke nadelige effecten:	Bij de vena punctie: volledig reversibel.<br>Bij antibiotica: volledig reversibel na eventueel aanvullende therapie.
Lichamelijke belasting (pijn, ongemak):	Pijn.<br>Mogelijk meer ziekteverschijnselen.
Psychische belasting (angst, stress):	Mogelijk stress voor venapunctie.

**Kenmerken onderzoekspopulatie**	
Kwetsbaarheid (ernstig zieken, kwetsbare ouderen, jonge kinderen):	Ernstig zieke patiënten die een ICU opname behoeven zijn uitgesloten van deelname.<br>Ouderen zullen wel deel mogen nemen aan de studie.

**Maatschappelijke risico's**	
Voor de proefpersoon (privacy, stigmatisering, uitsluiting van verzekering):	Geen.
Voor het onderzoek (maatschappelijk draagvlak, gevoeligheid van het onderzoek):	Mogelijk eerste stap in nieuwe behandelstrategie op populatieniveau. Geen controverse op maatschappelijk niveau.

**Risico's samenhangend met onderzoeksopzet en –uitvoering (risico op protocol violations)**	
Complexiteit van het protocol:	Eenduidig
Aantal te includeren proefpersonen:	468

**Conclusie risico-classificatie**	
Maak aan de hand van tabel 1 een keuze uit de opties:	☐ verwaarloosbaar risico
Geef een toelichting op de gemaakte keuze.	Toelichting:<br>Kleine kans op schade, die licht tot matig kan zijn echter wel reversibel is. Patiënten zullen bij toename van ziekteverschijnselen na eerder staken van antibiotica behandeld worden volgens het normale klinische pad. Dit houdt in dat zij vervolgens hetzelfde behandeld worden als patiënten die ervoor kiezen geen deel te nemen aan het onderzoek. Wij verwachten niet dat dit de opnameduur of (reguliere) behandelduur verlengd.

Tabel 1. Risicoclassificatie (verwaarloosbaar risico, matig risico, hoog risico) in relatie tot de kans op schade en de ernst van die schade.

Grootte van kans / Mate van schade	Lichte schade	Matige schade	Ernstige schade
Kleine kans	Verwaarloosbaar risico	Verwaarloosbaar risico	Matig risico
Matige kans	Verwaarloosbaar risico	Matig risico	Hoog risico
Grote kans	Matig risico	Hoog risico	Hoog risico

Appendix B: Definition of clinical and radiological responses[36]

Clinical response

Test of cure

Cure —resolution or improvement of symptoms and clinical signs related to pneumonia without the need for additional or alternative antibiotic therapy

Failure —persistence or progression of all signs and symptoms of the acute process after randomisation or the development of a new pulmonary or extrapulmonary respiratory tract infection, or the progression of abnormalities on chest radiograph after randomisation, or death due to pneumonia, or the inability to complete the study owing to adverse events

Indeterminate —patient receives less than 80% of the study drug for reasons other than clinical failure, a concomitant infection outside the respiratory tract requiring antibiotic treatment, lost to follow-up, or death unrelated to the primary diagnosis

Late follow-up

Cure —continued resolution or improvement of symptoms and clinical signs related to pneumonia without the need for additional or alternative antibiotic therapy

Recurrence —new or worsened signs and symptoms of pneumonia or another respiratory tract infection in a patient who was cured at the visit for test of cure

Indeterminate —lost to follow-up, a concomitant infection outside the respiratory tract requiring antibiotic treatment, or death unrelated to the primary diagnosis

Radiological response

Resolved — Areas of consolidation completely resolved

Improved — Areas of consolidation still exist but show evidence of clearing

Unchanged or worse — Areas of consolidation remain unchanged or show increased density

Appendix C: List of study procedures

	Day 1		Day 2		Day 3		Day 4		Day 5		Day 6		Day 7		Day 30	
	R	E	R	E	R	E	R	E	R	E	R	E	R	E	R	E
Blood tests	X	X					X			X		X		X		X
X-ray	X														X	
Sputum sample	X															X
Blood culture	X															
Nasopharyngeal swab		X														

Tabel 1: Study procedures. R = routine examination. E = extra diagnostic procedure related to the study.
